# Genomics Reveals the Origins of Historical Specimens

**DOI:** 10.1093/molbev/msab013

**Published:** 2021-01-27

**Authors:** Qian Cong, Jinhui Shen, Jing Zhang, Wenlin Li, Lisa N Kinch, John V Calhoun, Andrew D Warren, Nick V Grishin

**Affiliations:** 1 Eugene McDermott Center for Human Growth and Development, University of Texas Southwestern Medical Center, Dallas, TX, USA; 2 Department of Biophysics, University of Texas Southwestern Medical Center, Dallas, TX, USA; 3 Howard Hughes Medical Institute, University of Texas Southwestern Medical Center, Dallas, TX, USA; 4 McGuire Center for Lepidoptera and Biodiversity, Florida Museum of Natural History, University of Florida, Gainesville, FL, USA; 5 Department of Biochemistry, University of Texas Southwestern Medical Center, Dallas, TX, USA

**Keywords:** museomics, taxonomy, biodiversity, ancient DNA, geolocation

## Abstract

Centuries of zoological studies have amassed billions of specimens in collections worldwide. Genomics of these specimens promises to reinvigorate biodiversity research. However, because DNA degrades with age in historical specimens, it is a challenge to obtain genomic data for them and analyze degraded genomes. We developed experimental and computational protocols to overcome these challenges and applied our methods to resolve a series of long-standing controversies involving a group of butterflies. We deduced the geographical origins of several historical specimens of uncertain provenance that are at the heart of these debates. Here, genomics tackles one of the greatest problems in zoology: countless old specimens that serve as irreplaceable embodiments of species concepts cannot be confidently assigned to extant species or population due to the lack of diagnostic morphological features and clear documentation of the collection locality. The ability to determine where they were collected will resolve many on-going disputes. More broadly, we show the utility of applying genomics to historical museum specimens to delineate the boundaries of species and populations, and to hypothesize about genotypic determinants of phenotypic traits.

A study of every animal starts with its name, the creation of which is governed by a set of strict nomenclatural rules that have existed in one form or another for over a century. The current rules are regulated by the International Code of Zoological Nomenclature (ICZN 1999). The link between a name and a taxonomic unit (in our case a species or subspecies) is the type. Also known as the name-bearing type, this specimen, or a group of specimens, represents the objective standard of reference whereby the application of the name of that species or subspecies can be determined. For example, the type of *Homo sapiens* is Carl Linnaeus (Carl von Linné) (1707–1778), who is known as “the father of modern taxonomy” (Spamer 1999).

There are different kinds of types; the two of concern here are holotypes and lectotypes. A holotype is a single specimen that is designated as the name-bearing type of a species or subspecies when it is originally described. Only since the twentieth century has it been customary to designate a holotype, and when older descriptions unambiguously refer to a single type specimen, it is treated as the holotype. However, older descriptions were often based on a series of specimens, and it is sometimes discovered later that not all the specimens used to describe a particular animal represent the same species. In such instances, it is often necessary to formally select one of those specimens to represent the given name. Such a specimen, the lectotype, thereafter serves as the single, name-bearing type of that taxon. A lectotype is therefore designated after the establishment of a nominal taxon, sometimes many years later.

If a population is considered to be conspecific with a particular name-bearing type, then the name represented by that type is applied to the population. If two or more name-bearing types are deemed to be conspecific with one another, then the name that was published earliest generally has nomenclatural priority and is applied to the given population (though there are some technical exceptions to this rule). Therefore, determination of conspecificity is important for naming a population and resolving taxonomic conflicts, and such decisions are historically based on similarity in phenotype and locality. It is often challenging to distinguish closely related and phenotypically variable taxa, and thus the geographical place of capture of the type—the type locality—is very important when making these decisions. Locality information is particularly helpful in assigning a specimen to a subspecies, as different subspecies occupy different portions of the species’ range.

However, most descriptions of new species were published over a century ago, and their types are old and often lack details about their collection localities. Uncertain type localities, coupled with similar phenotypes between nominal taxa, can lead to heated debates about the application of given names, making it more difficult to resolve taxonomic conflicts (Calhoun 2015a, 2015c; Warren and Calhoun 2015; Scott 2016; Scott et al. 2018). In these instances, it is desirable to determine the locality for the associated name-bearing types. In addition to encoding a species’ phenotype, the DNA sequences of the types can reveal their geographical origin when compared against other specimens of the same species from known localities. Targeted sequencing of selected DNA markers, such as a segment from the mitochondrial gene encoding cytochrome C oxidase I (COI barcode), can identify most species with reasonable accuracy, as well as discover new cryptic species (DeSalle and Goldstein 2019) and associate historical types with present-day specimens (Janzen et al. 2017). However, these DNA markers may be insufficient in verifying a specimens’ collection locality, as different populations of the same species may not diverge within those genetic regions.

We devised a strategy to obtain genomic sequences of old type specimens and determine to which present-day populations they correspond. To solve a daunting taxonomic problem, we applied this strategy to the skipper butterfly *Hesperia comma* (originally *Papilio comma*) and its relatives. Taxonomically, it is the most important skipper, as this whole family of butterflies (Hesperiidae, the skippers) is typified by the genus *Hesperia*, and the type species of that genus is *H. comma*, which was described by Carl Linnaeus himself. Several taxonomic and biological mysteries surround these butterflies. First, the lectotype of *H. comma*, presumably collected in Sweden, lacks a locality label, and its American allopatric counterpart, *Hesperia colorado* (collected by noted naturalist Theodore Mead in 1871 and named as a species by Samuel Scudder in 1874), bears an imprecise locality label (Calhoun 2015a, 2015c). Where was the lectotype of *H. colorado* collected? Second, although originally proposed as a species, *H. colorado* has been frequently treated as a subspecies of *H. comma* (Scott 1986, 2016). Are American *comma*-like butterflies, such as *H. colorado* and presumed relatives, the same species as *comma* in Europe? Third, unusual among butterfly genera, *Hesperia* inhabits a wide range of elevations, from lowlands to alpine zones above 3,500 m. What are the genetic determinants of this elevational plasticity? Genomic data provide answers to all these questions.

One of the most intriguing taxonomic controversies involves the origin of the *H. colorado* lectotype specimen. A dozen *H. colorado* subspecies have been proposed (Pelham 2008), and each is recognizable by phenotype when a series of specimens representing a subspecies is compared against series of specimens of other subspecies. However, remarkable variation in wing patterns within each subspecies, and intergradation between them, increases the importance of locality in assigning a single specimen (such as the lectotype) to a subspecies. Due to the lack of a precise locality label, it was unclear which present-day population corresponded to the *H. colorado* lectotype. Some researchers (Scott 2016; Scott et al. 2018) suggested that the lectotype was from the subalpine zone, whereas others (Calhoun 2015a, 2015c; Warren and Calhoun 2015) argued that it was from the Arkansas River Basin. It was even proposed to be a hybrid of these two populations, and thus a poor choice for the name-bearing type (Scott 2016; Scott et al. 2018). Phenotypic traits without a link to a precise locality were insufficient to attribute the lectotype to any particular population. Such situations are common in zoology. In offering a general solution, we ventured to place the lectotype of *H. colorado* on the map using genomics. Determining its locality and genetic identity would thereby seal the fate of names given to several *H. colorado* subspecies. The population aligned with the *H. colorado* lectotype would therefore be identified as the nominotypical subspecies, *H. colorado colorado*.

## Results and Discussions

### *Hesperia colorado* Lectotype Mapped to Lake County in Colorado

**Fig. 1.**
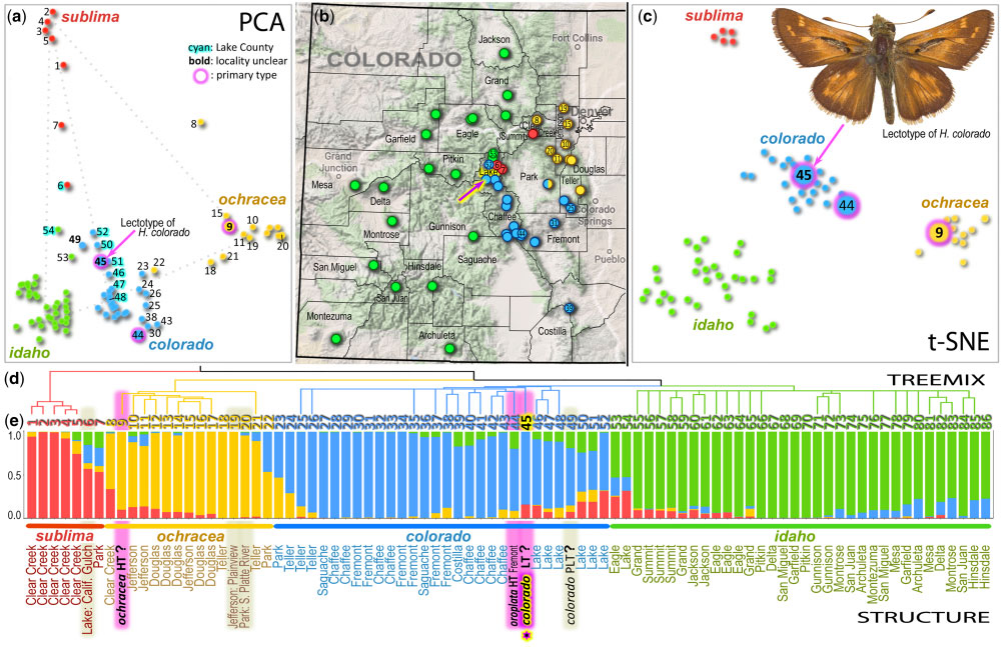
*Hesperia colorado* type specimen traced to Lake County, Colorado. Different methods consistently partition specimens into four populations (named subspecies): *sublima* (red), *ochracea* (orange), *colorado* (blue), and *idaho* (green). (*a*) Principal component analysis (PCA) of covariance between the SNPs in samples from Colorado using Eigensoft (Patterson et al. 2006). The 1st and 2nd components are shown. (*b*) Truncated map of Colorado showing specimen localities. Arrow points to the locality in Lake County we deduced for the *colorado* lectotype. (*c*) t-SNE (parameter: perplexity = 10) reduced the first ten PCA dimensions to two, revealing populations as clusters. (*d*) TREEMIX results showing the clustering and evolutionary history of specimens. (*e*) Population structure inferred by STRUCTURE, showing the proportion of each population’s features in each specimen. Names of taxa (*colorado*, *oroplata*, *ochracea*) are given for type specimens (HT holotype, LT lectotype, PLT paralectotype), and primary types are highlighted in magenta; other historical specimens are highlighted in gray; county names are given for other specimens (see other data in supplementary table S1, Supplementary Material online, referred to by the numbers from 1 to 86 given as leaves in the TREEMIX tree); a question mark after the name of a type indicates a previously uncertain collection locality that we identify here using genomic comparisons.

First, we sequenced and assembled a reference genome of *Hesperia colorado*. The genomic sequence was about 650 million base pairs, larger than most butterflies. Second, we overcame the challenge of sequencing a small, 150-year-old butterfly specimen without irreparably damaging it. This resulted in ∼25% complete nuclear and entire mitochondrial genomes of the *colorado* lectotype. Third, we obtained whole-genome shotgun sequences of 85 specimens across Colorado (supplementary table S1, Supplementary Material online). The reference assembly and sequencing data of specimens have been deposited in the NCBI database under BioProject PRJNA698011. The ages of these specimens varied from 150 years (from Mead’s same expedition) to recently collected. These sequences were mapped onto the reference genome and analyzed by a combination of population genetics tools. The result was unambiguous: the lectotype of *colorado* was geographically placed in an area about 15 km in diameter in Lake County, Colorado, and belongs to the Arkansas River Basin population (fig. 1, specimen no. 45).

Using Eigensoft (for principal components analysis, PCA), t-SNE, STRUCTURE, and TREEMIX analyses, we determined that *H. colorado* is represented by four major populations in Colorado (shown in blue, green, red, and yellow in fig. 1). Partitioning of these specimens into four populations is not only supported by the clustering of data points by PCA and t-SNE, it is also the most probable solution suggested by STRUCTURE. We tested a series of possible values for the number of populations (K) using STRUCTURE. The highest probability of observed data (supplementary fig. S13, Supplementary Material online), and the lowest number of hybrid specimens (supplementary fig. S14, Supplementary Material online), were achieved when we assumed four populations. These populations correspond to subspecies. The alpine subspecies, *H. c. sublima* is surrounded by three others along three major river basins: the Platte (*H. c. ochracea*), the Arkansas (*H. c. colorado*), and (the largest) the Colorado (tentatively assigned to *H. c. idaho*) (fig. 1*b*).

**Fig. 2.**
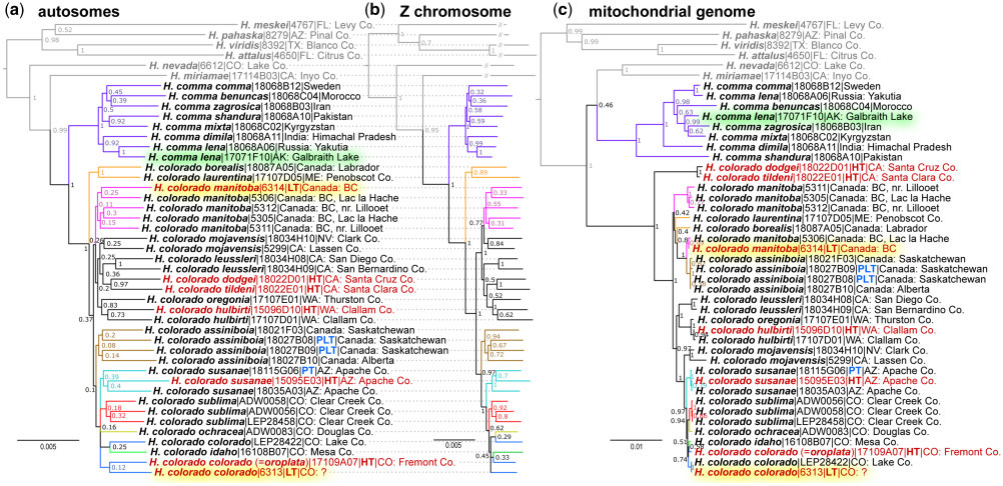
Phylogenetic analysis suggests that *Hesperia colorado* is a species distinct from *Hesperia comma*. The trees are based on concatenated genomic alignments from (*a*) autosomes, (*b*) Z chromosome, and (*c*) mitogenome. Name, voucher number, and general locality are given for each specimen (see supplementary table S1, Supplementary Material online, for additional data). *Hesperia* species outside the *comma* group are shown in gray. The trees are rooted with *Vernia verna* (NVG-18014H01, not shown). Names of primary type specimens are shown in red. The two types with previously uncertain localities are highlighted in yellow and an American *H. comma* specimen from Alaska is highlighted in green. Tree branches corresponding to clades discussed in this study are colored in different colors. The same colors are used for these clades (species, groups of subspecies, subspecies) in other figures.

These four subspecies intergrade at the boundaries of their ranges, forming hybrids (fig. 1*e*). Hybridization patterns are best revealed by the PCA analysis, where the hybrids line up between the centers of populations (along dotted lines in fig. 1*a*). The transition between the alpine (red) and Arkansas River Basin (blue) populations is in Lake County (cyan numbers in fig. 1*a*), where three subspecies meet and hybridize. The lectotype of *colorado* (no. 45 fig. 1*a*) is surrounded exclusively by the specimens from Lake County, implying where it was collected. Moreover, the mitogenome of the lectotype was a 100% match to a single specimen (fig. 2*c*), which was more recently collected near Twin Lakes in Lake County. Another of Mead’s specimens (no. 49, a paralectotype of *colorado*, fig. 1*a*), also collected during his 1871 expedition, maps to the same area. STRUCTURE (fig. 1*e*) reveals their genomic composition. Lake County specimens are characterized by having some *sublima* (red) and *idaho* (green) components, making them attributable to the narrow geographic zone of transition between these populations. Nevertheless, as t-SNE shows (fig. 1*c*), the lectotype of *colorado* clusters within the Arkansas River Basin population, and STRUCTURE reveals that only a minor fraction of its genome is of hybrid origin. A portion of this population was described by Scott (1981) as a subspecies named *H. c. oroplata* (no. 44 is its holotype), but the entire population represents the nominotypical subspecies, *H. c. colorado*, which is the older name, superseding *oroplata*, a younger name (Scott 1981).

As a control for our methods applied to historical specimens, we used three others (two collected by Mead) with less questionable localities (Calhoun 2015a, 2015c). These specimens (nos. 6, 19, and 20, fig. 1*a*) mapped accordingly with their presumed localities. Specimen no. 6, from near Leadville (Lake County), is indeed placed with the Lake County specimens, but closer to *sublima* as suggested by its genetic makeup revealed by STRUCTURE (fig. 1*e*). The other two specimens were attributed to *ochracea* in agreement with their phenotype and locality. Placing these controls builds the confidence in inferring localities of specimen numbers 45 and 49 by genomics. Moreover, historical evidence suggests that the lectotype of *colorado* was indeed collected in Lake County, as the specimen bears a label “7–13” (July 13). According to Mead’s personal journal, he was at Twin Lakes, Lake County, on that date (Calhoun 2015c). The lectotype of yet another subspecies, *H. colorado manitoba* (originally *Pamphila manitoba* Scudder), had a similarly controversial type locality. Our results place it next to a specimen from Lac la Hache in British Columbia (fig. 2*a–c*), in accordance with its label data, thereby validating the type locality of *manitoba* (Calhoun 2015b). We think that our success in deducing such localities is due to the fact that *Hesperia* are nonmigratory, local butterflies that form well-diverged populations.

Topology of the nuclear genomic trees follows the geographic distribution of these populations: those that are close on the map tend to cluster in the trees. For example, all specimens from Colorado, including the *colorado* lectotype, form a clade, which includes the nearby populations from eastern Arizona. However, the mitogenome tree (fig. 2*c*) is incongruent with the nuclear genome trees, revealing a history of mitochondria different from that of nuclear genomes, a phenomenon commonly observed in closely related populations and species (Cong, Shen, Borek, et al. 2017). Nevertheless, all three trees place both the *manitoba* and *colorado* type specimens in agreement with their collection localities (Calhoun 2015b, 2015c), contrary to the speculations of Scott (Scott 2016; Scott et al. 2018). The trees also support the distinction between Old World *comma* (also found in Alaska) and the North American species *H. colorado.*

### *Hesperia colorado* Is a Species Distinct from *Hesperia comma*

Many American species have similar-looking counterparts in Europe and Asia, posing a question about whether we should regard them as the same species. Phylogenetic trees of three different genomic regions (autosomes, Z-chromosome, and mitochondria) consistently reveal a deep split between American *Hesperia colorado* and the Old World *Hesperia comma* (fig. 2), strongly suggesting that they are not conspecific. The genome-wide Fixation index (*Fst*) (Holsinger and Weir 2009) between *comma* and *colorado* is about 0.5, a value typical for different species of animals and plants (Hey and Pinho 2012). In addition, we computed the fixation index and the level of gene flow for Z-linked genes, which are able to distinguish pairs of species from conspecific populations based on the study of butterfly speciation across central TX suture zone (Cong et al. 2019). The level of divergence between *H. colorado* and *H. comma* reflected by these statistics are comparable to other pairs of species, and very different from values computed for conspecific populations (supplementary fig. S17, Supplementary Material online). Finally, we used coalescent-based species delimitation method, Bayesian Phylogenetic and Phylogeography (BPP), to identify independently evolving lineages and performed 100 simulations based on randomly selected loci (20 loci at a time) under the multispecies coalescent model (Yang 2015). All the simulations suggested that the probability for *H. colorado* and *H. comma* to be independently evolving species is 100%.

Despite its broad distribution, populations of *comma* from Europe, Africa, and Asia are much closer to each other than any are to Nearctic *colorado*. Majority of the *comma*-like specimens we sequenced from the United States and Canada are *colorado*, with only one exception. A specimen from northern Alaska turned out to be true *comma*, both phenotypically and by genomic data. It is the most similar to specimens from northeastern Russia across the Bering Strait ( of proteins that differ the most between the two species reveals the prevalence of nuclear-encoded mitochondrial respiratory chain components, which is consistent with the profound divergence (about 3%) in the mitogenomes. Furthermore, the two species show divergence in circadian rhythm, DNA, and histone methylation, which may contribute to the reproductive barrier between *comma* and *colorado*.

### Putative Adaptations to High Elevation in *Hesperia colorado sublima*

The high-elevation subspecies, *H. c. sublima*, contains the highest number of unique SNPs, two to four times higher than other low-elevation populations (supplementary fig. S18, Supplementary Material online). There are two possible reasons for this observation: 1) *sublima* specimens show the lowest level of population polymorphism (π) (supplementary fig. S19, Supplementary Material online), indicating a smaller effective population size. Smaller population size may allow *sublima* to accumulate more unique SNPs as a result of stronger genetic drifts on neutral mutations or rare ancestral alleles; and 2) *sublima* shows higher level of nonsynonymous mutation rate (supplementary fig. S20, Supplementary Material online), indicating stronger positive selection in this mountain-top population.

**Fig. 3.**
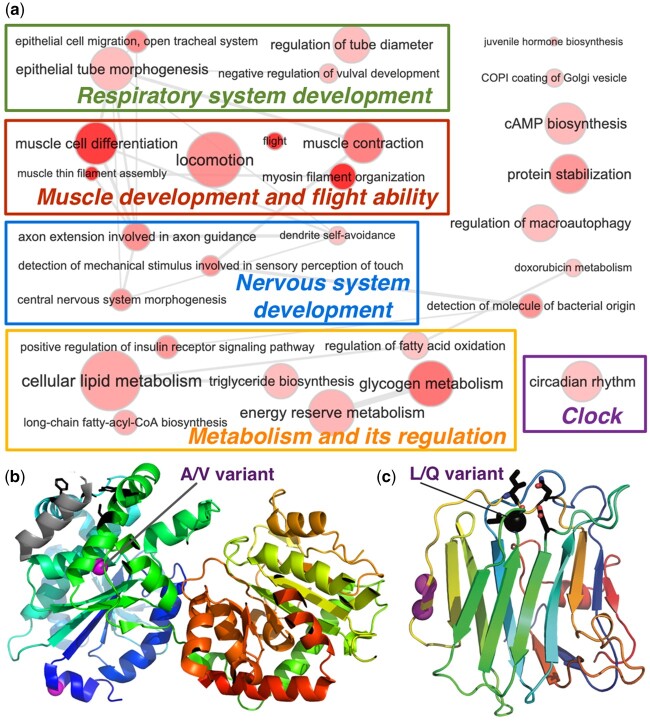
Molecular adaptation to high elevation in *Hesperia colorado sublima*. (*a*) Biological processes related to high-elevation adaptation genes revealed by Gene Ontology (GO) terms. The size of a circle correlates with the number of proteins in the genome associated with that GO term and its color indicates the significance (darker color corresponds to lower *P* value) of a GO term's enrichment among proteins showing high-elevation adaptation. (*b*) 3D structure model of glycogen synthase (template PDB:4QLB). A variant in *sublima* (from A to V) is near the functional sites (black sticks) mediating its interaction with glycogenin. (*c*) 3D structure model (template PDB:3POY) of motor neuron guidance factor *trol* with a variant (black sphere, L in *sublima*, and Q in low-elevation populations) near the Ca2+ binding site. Other variants are shown as magenta spheres.

We applied BayeScan (Foll and Gaggiotti 2008) to identify positions showing significant impact of positive selection in *sublima* by comparing the amino acid frequencies in proteins between *sublima* and lower-elevation subspecies. We found 112 positively selected positions from 72 proteins (False Discovery Rate for multiple statistical tests <10%) (supplementary table S2, Supplementary Material online). These proteins function in muscle development and flight ability, respiratory and nervous systems development, lipid/glycogen metabolism, and its regulation (fig. 3*a*). We further identified ten proteins under significant positive selection (adaptive evolution) using McDonald–Kreitman tests (McDonald and Kreitman 1991). These genes overlap significantly with those identified using allele-frequency based method, and they again mostly function in muscle development, flight, and glycogen metabolism (supplementary table S2, Supplementary Material online).

The adaptation of *H. c. sublima* to high elevation should have a longer history (∼100,000 ya) than high-elevation adapted human populations, which dated up to 45,000 ya (Ossendorf et al. 2019). In addition, *sublima* has a 1- to 2-year generation span, allowing it to evolve faster than humans through germ-line mutation and genetic drifts. Human adaptations are largely restricted to variations in hypoxia-inducible factors, which upregulate glycogen and ATP synthesis under hypoxic conditions. Similarly, in *sublima*, sites predicted to undergo positive selection map to regulators of insulin pathway, glycogen synthase, and phosphorylase, in addition to metabolic enzymes involved in oxidative reduction reactions. These variations may reprogram *sublima* metabolism to ensure sufficient energy production with reduced oxygen. One single amino acid variant (SAV) of *sublima* locates in the helix of glycogen synthase that mediates its interaction with glycogenin (fig. 3*b*). The interaction between the glycogen synthase and glycogenin is crucial (Zeqiraj et al. 2014) for the synthesis of glycogen polymers, and the *sublima*-specific SAV affecting this interaction may lead to more efficient glycogen production.

Insects lack the dedicated oxygen-carrying blood cells of vertebrates, and a network of tracheal tubes directly delivers oxygen throughout the body. Similar to humans growing more capillaries in muscle with exercise at high elevation (Mathieu-Costello 2001), *sublima*-specific variations in factors governing the development of epithelial tubes may promote elaboration of tracheal tubes (green box in fig. 3*a*). Finally, the most significantly enriched function associated with the putative high-elevation adaptation proteins are all related to muscle development and flight ability (supplementary table S3, Supplementary Material online). For example, the motor neuron axon guidance factor *trol* contains a *sublima*-specific SAV near its calcium binding site: in *sublima*, hydrophobic Leucine replaces hydrophilic Glutamine of lower elevation populations (fig. 3*b*). Strong fliers, including swallowtails (Papilionidae), skippers (Hesperiidae), and monarch butterflies (*Danaus plexippus*, Nymphalidae), all have hydrophobic residues in this position, whereas in weak fliers, such as the cabbage white (*Pieris rapae*, Pieridae) and the genus *Eumaeus* (Lycaenidae), this residue is hydrophilic (supplementary fig. S22, Supplementary Material online). These changes might make *sublima* more able to fly in the stronger winds associated with mountaintops.

### Genetic Basis for Paler Appearance of *Hesperia colorado ochracea*

**Fig. 4.**
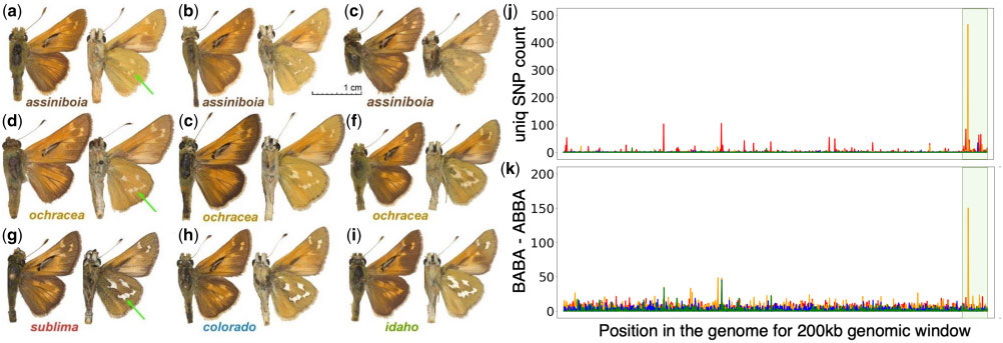
Similarity in wing pattern between *Hesperia colorado ochracea* and *H. c. assiniboia* likely caused by the introgression of a 200-kb Z-linked genomic region. Wing pattern variation in (*a–c*) *assiniboia* and (*d–f*) *ochracea* specimens; (*g–i*) typical specimens of other *Hesperia* populations in Colorado. Green arrows denote pale spots on the hindwing differing between populations. Voucher numbers for (*a–i*) are NVG-18027B08, NVG-18027B09, NVG-18027B10, NVG-15111B01, NVG-5533, NVG-16108A05, NVG-5532, NVG-16108C02, and NVG-16108B04, respectively; see supplementary table S1, Supplementary Material online, for additional data. (*j*) Number of unique SNPs in different populations in 200-kb windows throughout the genome. (*k*) Introgression from *assiniboia* to four *Hesperia* populations in Colorado identified using the ABBA-BABA test. The *y* axis shows the difference between the number of positions with the pattern ABBA and the number of positions with the pattern BABA in a 200-kb genomic window (negatives omitted). The *x* axis on both plots is the position of the window in the genome (concatenated scaffolds). Z chromosome scaffolds are placed last (highlighted pale olive). Counts are colored by taxon: *sublima* (red), *colorado* (blue), *idaho* (green), and *ochracea* (orange).

Of all populations of *H. colorado* in Colorado, *ochracea* is perhaps most recognizable due to its overall paler coloration and poorly defined white spots on the ventral hindwing. We counted the number of unique SNPs in each subspecies in genomic windows of 10 kb. We found that 23% of the unique SNPs in *ochracea* are concentrated in a 200-kb region (0.03% of the entire genome) on the Z-chromosome out of the 609-Mb genome (fig. 4). This unusually high density of SNPs suggests introgression from some population outside of Colorado, rather than gradual evolution by point mutations. We constructed a phylogenetic tree of all sequenced *Hesperia* specimens using this 200-kb region. *Hesperia c. ochracea* groups with populations associated with the name *assiniboia*, a northern butterfly of the Central Plains, which is recognized as a subspecies of *H. colorado* or a distinct species. Therefore, we hypothesized that this 200-kb region is introgressed from *assiniboia*, and we carried out ABBA-BABA analysis to test our hypothesis.

ABBA-BABA tests (Martin et al. 2015) indeed support that this 200-kb region was introgressed (*P* value <6e-66) from *assiniboia*. Because the wing patterns of *ochracea* resemble *assiniboia* (fig. 4*a–f*), it is likely that this introgressed region is inducing *assiniboia-*like wing patterns in *ochracea.* Proteins encoded by this region include *Shank3*, a regulator of *Wnt* signaling pathway that functions by modulating internalization of the *Wnt* receptor *Fz2* (Harris et al. 2016). The *Wnt* pathway has been implicated in wing patterning (Martin et al. 2012; Martin and Reed 2014; Mazo-Vargas et al. 2017), and *Wnt* receptor *Fz2* is expressed in developing wings (Hanly et al. 2019), suggesting a possible role of *Shank3* in wing pattern formation.

## Materials and Methods

A detailed version of our experimental and computational methods, as well as important intermediate results and configuration files for the programs we used, are provided in the supplemental materials.

### DNA Library Preparation and Sequencing

Specimens used in this project were collected in the field (and stored in RNAlater or EtOH) or borrowed from collections listed in the Acknowledgments. The collection year of specimens ranged from 1871 to 2016 (see supplementary table S1, Supplementary Material online, for complete specimen data). A piece of thoracic tissue from fresh specimens, and either the abdomen or a leg from pinned museum specimens, were used for DNA extraction and genomic library preparation according to our protocols developed previously (Cong et al. 2015; Li et al. 2019; Zhang, Cong, Rex, et al. 2019; Zhang, Cong, Shen, et al. 2019). Libraries were sequenced for 150 bp from both ends targeting 5–10× coverage.

Additional precautions were taken for historical type specimens over 100 years old: 1) instead of processing them in plates of 96 specimens together, we processed them one by one in individual tubes to avoid cross-contamination; 2) we replaced the buffer in Ampure XP beads with solution containing 30% PEG, and we used 3× volume of Ampure XP beads (with 30% PEG) to purify the adapter-ligated DNA fragments, allowing the beads to bind DNA fragments of smaller size; 3) we treated DNA with NEB PreCR repair enzymes before PCR; and 4) we performed size selection of DNA after PCR amplification using DNA gel instead of beads, as the DNA fragments we needed were not much longer than adapter dimers.

### Reference Genome Assembly and Annotation

We used paired-end libraries and mate-pair libraries of 2, 5, and 10 kb to assemble a reference genome of *Hesperia colorado* from a single wild-collected specimen (ADW0057). After removing low-quality portions and adapters from the reads, we corrected errors in the reads using QUAKE and assembled the genome using Platanus. After identifying and masking repeats with RepeatModeller and RepeatMasker, we annotated the genome using three approaches: RNA-seq based, homology-based, and de novo gene prediction.

As references for homology-based annotation, we used protein sets from other species of Lepidoptera: *Papilio machaon* (Paplionidae) (Li et al. 2015), *Pieris rapae* (Shen et al. 2016), *Calycopis cecrops* (Lycaenidae) (Cong et al. 2016), *Calephelis nemesis* (Riodinidae) (Cong, Shen, Li, et al. 2017), *Danaus plexippus* (Zhan et al. 2011), *Cecropterus lyciades* (Hesperiidae) (Shen et al. 2017), and *Bombyx mori* (Bombycidae) (Kawamoto et al. 2019), as well as a species of Diptera, *Drosophila melanogaster* (dos Santos et al. 2015). The reference protein sets were aligned to the genome assembly using exonerate (Slater and Birney 2005). We aligned the RNA-seq reads to the reference genome using TopHat (Trapnell et al. 2009), and derived transcript-based annotations using Cufflinks (Trapnell et al. 2010). Three methods were used to obtain de novode novo gene annotations: Augustus (Stanke et al. 2004), GeneMark_ES (Lomsadze et al. 2005), and SNAP (Korf 2004). Finally, annotations by different approaches were combined in EvidenceModeler (Haas et al. 2008) to obtain their consensus as the final gene predictions.

We predicted the functions of these proteins by finding the closest homologs in Flybase (Thurmond et al. 2019) and Swissprot (UniProt Consortium 2019) using BlastP (Altschul et al. 1990) (*E*-value <0.00001) and transferred the Gene Ontology (GO) (Gene Ontology Consortium 2015) terms and function annotations. Z chromosome scaffolds were found as those containing Z chromosome proteins in the Lepidoptera genus *Heliconius* (Nymphalidae) (Davey et al. 2016).

### Genomic Sequence Assembly

We assembled the genomes of other specimens by mapping the reads to the reference and SNP calling. We processed the sequencing reads using Trimmomatic (Bolger et al. 2014) and PEAR (Zhang et al. 2014). The resulting reads of each specimen were mapped to the reference genome using BWA (Li and Durbin 2009). Since many specimens were more than a century old and their DNA could be contaminated, we developed protocols to clean up the alignments by the consistency between reads of a particular specimen, and between these reads and the reference genome.

We performed SNP calling for each specimen using samtools (Li et al. 2009). For PCA (Price et al. 2006), t-SNE (van der Maaten and Hinton 2008), and STRUCTURE (Pritchard et al. 2000) analyses, we derived the genomic sequence of each specimen by taking the SNPs called at positions that are covered by at least two different reads and filling the remaining positions with gaps. To prepare the input for TREEMIX (Pickrell and Pritchard 2012), we did not perform SNP calling. Instead, we recorded the frequency of each nucleotide in the sequencing reads at each position. To prepare the input for phylogenetic reconstruction, we obtained the dominant (frequency > 0.6) nucleotide at each position.

### Analyzing Genomic Data of *Hesperia* Specimens Using Population Genetic Tools

A unique challenge of this study was to properly analyze the historical museum specimens whose genomes are highly incomplete (supplementary table S4, Supplementary Material online). Out of the 86 *Hesperia colorado* specimens, 14 were less than 50% complete (NVG-5304, NVG-5533, NVG-6313, NVG-6705, NVG-6706, NVG-6708, NVG-7574, NVG-7575, NVG-15111B01, NVG-15111B02, NVG-16108A06, NVG-16108A07, NVG-16108A08, and NVG-16108C04). Although it may be possible to sequence more of the same specimens, thus increasing genomic completeness, it significantly increases the costs of the project and we opted to solve the problem computationally with existing data. Population genetic tools such as Eigensoft (Price et al. 2006) and TREEMIX (Pickrell and Pritchard 2012) are not adapted to handle data sets with many gaps (missing data), but removal of positions that contain gaps drastically decreases the amount of data that can be used in the analysis. We solved the problem by performing analyses with only well-covered specimens (“backbone” specimens) first, then we added the remaining poorly covered specimens (“target” specimens) one by one to the obtained confident “backbone” to determine their placement.

To prepare the input files for Eigensoft and STRUCTURE (Patterson et al. 2006), we first processed the alignments to remove positions with gap ratios above a certain cutoff: four gap ratio cutoffs were used here: 0.1, 0.15, 0.2, and 0.25. We next selected confident biallelic loci from the alignment. We considered positions with two possible nucleotides and required each nucleotide to be present in at least three specimens, as low-frequency SNPs may represent errors in sequencing or random damage in DNA of a museum specimen. We further selected representative positions among linked loci using plink (Purcell et al. 2007). We counted the number of positions after processing under different gap ratio cutoffs, and we selected the cutoff resulting in 50,000–100,000 positions for each input alignment.

We first obtained the PCA result for the 72 well-covered specimens using Eigensoft. We excluded the hybrid specimens (black dots in supplementary fig. S3, Supplementary Material online) and used the rest as “backbone” for PCA analysis (supplementary fig. S4, Supplementary Material online). We performed PCA for each “target” specimen with the “backbone” specimens and the results are given in supplementray figures S5–S7, Supplementary Material online. In order to visualize all the “target” specimens together with the backbone specimens, we combined the PCA projections for each specimen with the overall PCA projection containing only well-covered specimens. We considered each of the PCA results displayed in supplementary figures S3–S7, Supplementary Material online, as a 2D image and found the transformations that make the coordinates of “backbone” specimens (present in all the images) superimposed between different images with the minimal root mean square distance. We allowed translation, rotation, and rescaling in these transformations, and the python script to find the best transformation is provided in the supplemental methods.

To summarize information from more (up to ten) Principle Components (PCs), we applied t-SNE (van der Maaten and Hinton 2008) to process the outputs of Eigensoft. Because t-SNE does not work well with hybrids that cannot be clustered into any group, we excluded hybrids for such analyses. T-SNE needs a parameter, namely perplexity, to indicate the expected size of each cluster. We found that perplexity of 6 or 7 gave results that were consistent with geographical locality and STRUCTURE. T-SNE results for the “backbone” specimens only and the “backbone” specimens with each “target” specimen are shown in supplementary figures S9–S11, Supplementary Material online. To visualize the t-SNE results for all the “target” specimens together, we merged the panels in supplementary figures S10 and S11, Supplementary Material online, to supplementary figure S9, Supplementary Material online, by placing each “target” at the coordinate that can preserve the relative distances between this “target” specimen and the four subspecies as those in supplementary figures S10 and S11, Supplementary Material online.

We deduced the population structure for the 72 well-covered species using STRUCTURE and selected a set of relatively pure “reference” specimens (four to six specimens per subspecies, see supplementary table S4, Supplementary Material online). We then analyzed each of the 14 “target” specimens separately with the “reference” specimens, where we provided population labels for these “references.” From each alignment, we randomly sampled 5 sets of inputs for STRUCTURE, each consisting of 50,000 positions. For each set of 50,000 positions, we ran STRUCTURE assuming 2, 3, 4, 5, 6 populations (*K* = 2, 3, 4, 5, 6) with ten replicates for each value of K. Each replicate was initiated with a different random seed. Therefore, for each value of K, we have 50 STRUCTURE results: 5 data sets and ten replicates for each data set. We inspected the STRUCTURE output with the highest “Estimated Ln Prob” from the STRUCTURE output for each K (supplementary fig. S12, Supplementary Material online). At *K* = 4, STRUCTURE produces results with the highest probability (supplementary fig. S13, Supplementary Material online).

In our experience, TREEMIX is even less tolerant to gaps in the input genotype data than previously described tools. Therefore, we eliminated all the gaps in the TREEMIX input and used only the non-hybrid specimens with gap ratio less than 25% as “backbone” specimens to place the “target” specimens. A total of 51 specimens representing all four populations were selected (supplementary table S4, Supplementary Material online), and each of the remaining specimens was added to the “backbone” one at a time. We obtained more than 200,000 positions in the alignment with the 51 “backbone” specimens. In order to obtain the support for each node, we generated 100 sets of randomly sampled 100,000 positions from each alignment and summarized the resulting trees by sumtrees.py (https://dendropy.org/programs/sumtrees.html, last accessed January 29, 2021).

### Phylogeny and Species Delimitation

We performed phylogenetic analysis using 45 representatives of various subspecies of *Hesperia colorado* and *Hesperia comma* over a wide range (fig. 2). Specimens from diverse localities and type specimens were preferred in our selection of representatives. One *Vernia verna* (Hesperiidae; formerly in *Pompeius*) specimen (NVG-18014H01) was added as the outgroup to root the tree. We obtained 100 random samples of 50,000 positions from the alignment of autosomal regions. For each sampled alignment, we performed phylogenetic analysis by IQ-TREE with the best substitution model inferred by the program, TVM+F+R4 (Nguyen et al. 2015). We used sumtrees.py to derive a consensus of the trees from 100 samples. Similarly, we did phylogenetic analysis on each of the 100 random samples of 50,000 positions from Z-chromosome alignment with IQ-TREE (model: TVM+F+R3). Again, we used sumtree.py to get a consensus between the 100 trees on different samples. For the mitochondrial genomes, we used bootstrap to generate 100 replicates and applied IQ-TREE on each replicate with the substitution model TIM2+F+R3.

To test whether *H. colorado* and *H. comma* should be treated as different species, we used coalescent-based species delimitation method, Bayesian Phylogenetic & Phylogeography (BPP) (Flouri et al. 2018). We selected one representative (the one with the least amount of gaps) for each of the *H. colorado* subspecies and each of the *H. comma* subspecies shown in figure 2. In addition, *Hesperia nevada* and *Hesperia viridis* were added as the more distantly related species and the outgroup for BPP analysis. We used gap-free positions and identified genomic segments (loci) consisting of 1,000–2,000 gap-free positions that were separated from each other by at least 10 kb in the genome. We randomly selected ten loci for each BPP simulation (speciestree = 1, speciesdelimitation = 1), and a total of 100 simulations were performed.

### Possible High Elevation Adaptation in *H. c. sublima*

We selected five representative specimens for each subspecies (*sublima*, *ochracea*, *colorado*, and *idaho*), and identified the uniquely frequent SNPs for each subspecies (frequency >75%, and absent in other subspecies). We used BayeScan (Foll and Gaggiotti 2008) to detect candidate loci under positive selection based on frequencies of amino acids in the high-elevation subspecies and other low-elevation subspecies. We identified enriched Gene Ontology (GO) (Gene Ontology Consortium 2008) terms associated with proteins containing positions under positive selection by binomial tests (*m* = the number of proteins with positively selected positions that were associated with this GO term, *N* = number of proteins containing positions predicted to be undergoing positive selection, *P* = the probability for this GO term to be associated with any protein). GO terms with *P* values lower than 0.01 were visualized using REVIGO (Supek et al. 2011).

We also identified the positively selected genes in *H. c. sublima* using McDonald–Kreitman (MK) tests (McDonald and Kreitman 1991). Proteins that revealed significant impact of positive selection both by the allele frequency and the MK tests were studied manually. We searched for homologous 3D structures of these proteins in the Protein Data Bank (Burley et al. 2017) using BlastP (Altschul et al. 1990) and interpreted the effect of the *sublima*-specific SAVs in the context of the structure templates and literature about their function.

### ABBA–BABA Test for Introgression in *H. c. ochracea*

ABBA–BABA test requires 4 taxa following a tree topology ((S1, S2), S3), O; where S1 and S2 are closely related, S3 is more distant and O is the outgroup. We carried out ABBA–BABA tests in the following four setups: 1) ((*H. c. sublima*, *H. c. ochracea* + *H. c. colorado* + *H. c. idaho*), *H. c. assiniboia*), *H. comma*); 2) ((*H. c. ochracea*, *H. c. sublima* + *H. c. colorado* + *H. c. idaho*), *H. c. assiniboia*), *H. comma*); 3) ((*H. c. colorado*, *H. c. sublima* + *H. c. ochracea* + *H. c. idaho*), *H. c. assiniboia*), *H. comma*); 4) ((*H. c. idaho*, *H. c. sublima* + *H. c. ochracea* + *H. c. colorado*), *H. c. assiniboia*), *H. comma*). We divided the genome into 200-kb windows. For each 200-kb window in each setup, we counted the number of positions following the pattern of ABBA or BABA in taxa S1, S2, S3, and O. We summed up the numbers we got for each position in the 200-kb window to obtain the total number of ABBA positions and BABA positions, respectively. The difference between the number of BABA positions and the number of ABBA positions is expected to be 0 if there is no introgression. A value significantly larger than 0 suggests introgression from *H. c. assiniboia.*

## Supplementary Material

Supplementary data are available at *Molecular Biology and Evolution* online.

## Supplementary Material

msab013_Supplementary_Data
