# Supplementary material for: Genomics Reveals the Origins of Historical Specimens
Source: Mol Biol Evol. 2021 Jan 27;38(5):2166–76. doi: 10.1093/molbev/msab013 (PMC8097301; doi:10.1093/molbev/msab013)
Supplement: msab013_Supplementary_Data [file msab013_supplementary_data.zip › Hesperia_supplement_revised.pdf]

Table of Contents

Extended methods 2

Section I: Genome Sequencing, Assembly and Annotation 2

I.1. DNA extraction, library preparation and sequencing 2

I.2. Protocol for gDNA extraction and library preparation for historical specimens 2

I.3. Reference genome assembly and annotation 7

I.4. Assembling genomic sequences 8

Section II: Population Genetic study of *Hesperia colorado* populations in Colorado State of the United States. 9

II.1. Handling specimens with poor data quality 9

II.2. Alignment processing 11

II.3. Decomposing genomic data with Principal Component Analysis (PCA) and clustering the specimens with t-SNE 12

II.4. Analyzing the population structure using STRUCTURE (Pritchard, et al. 2000) 24

II.5. Analyzing the population structure using TREEMIX 30

Section III: Phylogeny of *Hesperia colorado* and its sister species 32

Section IV: Correlating the Genotype with Phenotype 34

IV.1. Investigating the high elevation adaptation in *H. c. sublima* 34

IV.2. Genetic basis for the unique phenotype of *H. c. ochracea* 38

References 39

Extended methods

Section I: Genome Sequencing, Assembly and Annotation

I.1. DNA extraction, library preparation and sequencing

Materials used in this study were either fresh specimens collected in the field and preserved in *RNAlater* solution (wings and genitalia were stored in glassine envelopes), or older specimens pinned in collections. For specimens preserved in *RNAlater*, DNA was extracted from several pieces of tissue including the head, thorax and abdomen. For most pinned specimens, including the type specimens, we extracted DNA from the abdomen. For specimens with previously dissected genitalia, we used pieces of tissue from the thorax accessed from the abdomen attachment site. For some specimens, we took a leg for genomic DNA extraction. We prepared paired-end libraries using NEB Ultra II DNA library prep kit following our published protocols (Li, et al. 2019; Zhang, et al. 2019). Specimen ADW0057 was used to obtain the reference genome, and we made paired-end libraries at 250 bp and 500 bp, as well as mate pair libraries at 2 kb, 5 kb, and 10 kb for it. The protocols for making mate pair libraries were detailed in our previous publication (Cong, et al. 2015b).

For the reference genome, we obtained about 30 Gb, 15 Gb, 10 Gb, 6 Gb, and 4 Gb data for the 250 bp, 500 bp, 2 kb, 5 kb, and 10 kb libraries. For all other specimens, we targeted to obtain 3 Gb data per specimen. The mate pair libraries and the 500 bp paired-end library (for reference genome) were sequenced for 150 bp from both ends using illumina Hiseq 2500 platform. All other libraries were sequenced by Illumina Hiseq X10 to obtain 150 bp from both ends.

I.2. Protocol for gDNA extraction and library preparation for historical specimens

Type specimens used in the study were handled with extra precaution for the following reasons. (1) The type specimens are invaluable, and we would like to minimize the additional damage to the specimens due to the gDNA extraction process. (2) Type specimens are frequently around 100 years old and the amount of gDNA remaining in each specimen is usually very low. Therefore, we do not process historical specimens with any fresh specimens to avoid possible contamination. We normally extract gDNA and prepare libraries in batches of 96 samples on 96-well plates. However, for historical type specimens, we process them in small batches of 4-8 specimens of similar age and in individual tubes. (3) The gDNA in type specimens are frequently very short (less than 50 bp), and thus normal DNA purification and size selection procedures may fail to retain it. Our protocol to handle type specimens is outlined below.

I.2.1 Protocol for gDNA extraction

A. Before start

A.1. Thoroughly clean the work space, pipettor centrifuge, thermocycler with DNA AWAY (Thermo Scientific, 7010).

A.2. Set water bath at 56°C.

A.3. Make sure that 100% EtOH has been added to buffer B5 (MACHEREY-NAGEL, 740921).

A.4. Preheat the buffer BE (MACHEREY-NAGEL, 740306.100) to 56 °C, can distribute buffer BE in 1.5 ml tubes and let it stay on the heater.

A.5. Make sure that all the pipette tips are with filters (such as Rainin RT LTS 1000 μl FL 768A/8). All the liquid transferring should be done using pipette tips with filters to avoid contamination.

A.6. Make sure to use the clean reagents reserved for historical specimens or open new reagents to avoid possible contamination from reagents.

B. Lyse the sample

B.1. Carefully crack the abdomen off a specimen and place it in a 1.5 ml Eppendorf LoBind tube. Wash it with 70% alcohol to remove possible contaminants on the surface.

B.2. Add 180 μl buffer T1 (MACHEREY-NAGEL, 740940.25) and 20 μl proteinase K (MACHEREY-NAGEL, 740506) to the tube.

B.3. Invert the tube to mix, incubate at 56°C for 2-3h.

B.4. Squeeze the abdomen with 200 µl pipet tips gently. May push on the abdomen with the side of tips to squeeze the lysis buffer out and in gently.

B.5. Invert the tube to mix. Continue with digestion at 56°C for overnight.

B.6. If the abdomen does not seem to be fully digested (become soft), add 20 µl of Proteinase K (MACHEREY-NAGEL, 740506) and mix once again in the middle of digestion.

B.7. When the tissue becomes very soft, gently move the tissue out from the digestion tube and put it in another tube. Store the tube in freezer temporarily. If gDNA extraction from a sample succeeds, the post-digestion abdomen will be used to dissect genitalia.

B.8. Centrifuge the digestion tube at 10,000g for 1 min in room temperature, transfer supernatant to a new tube.

B.9. Add 200μl B3 (MACHEREY-NAGEL, 740920) and mix well by pipetting up and down.

B.10. incubate at 56°C for 2h. (Treatment with RNase A is not needed for old samples)

C. Bind the DNA

C.1. Add 210μl 100% EtOH and mix well by pipetting up and down.

C.2. Load the lysate onto a Zymo-Spin I column (ZYMO RESEARCH, C1003-50).

C.3. Centrifuge for 1 minute at 10,000g until sample mixtures have been completely filtered.

C.4. Discard the flow-through.

D. Wash the DNA

D.1. Add 300 μl wash buffer BW (MACHEREY-NAGEL, 740922), wash the wall of the column when adding the buffer.

D.2. Centrifuge for 1 minute at 10,000g until sample mixtures have been completely filtered.

D.3. Add 400 μl wash buffer B5 (MACHEREY-NAGEL, 740921), wash the wall of the column when adding the buffer.

D.4. Centrifuge for 1 minute at 10,000g until sample mixtures have been completely filtered.

D.5. Discard the flow-through.

D.6. Centrifuge for 2 minutes at 10,000g to dry the column.

E. Elute the DNA

E.1. Add 15 μl buffer BE (MACHEREY-NAGEL, 740306.100) right on the matrix in the column.

E.2. Incubate for 2 min.

E.3. Centrifuge for 1 minutes at 10,000g until sample mixtures have been completely filtered.

E.4. Add 15 μl buffer BE (MACHEREY-NAGEL, 740306.100) right on the matrix in the column.
E.5. Incubate for 2 min.
E.6. Centrifuge for 2 minutes at 10,000g until sample mixtures have been completely filtered.
E.7. A total volume of 28 μl gDNA solution is typically collected.

F. Quantify the DNA (optional)

We normally measure DNA concentration with 1 μl extracted gDNA using QuantiFluor dsDNA System flowing manufactory's protocol. However, for the historical type specimens, to preserve the gDNA as much as possible, we skipped this step. The concentration of gDNA extracted from the abdomen of historical specimens (around 100 years old) is usually on the magnitude of 0.01 ng/μl or 0.1 ng/μl (in 28μl).

I.2.1 Protocol for sequencing library preparation

A. Prepare Ampure XP beads in buffer with 30% PEG

Rationale: Ampure XP beads are able to bind shorter DNA in higher concentration of PEG. To increase the binding affinity of the beads to the short DNA in historical specimens, we replace the original solution of Ampure XP beads with similar solution containing 30% PEG (higher than the original beads solution). In addition, we use 3X volume of Ampure XP beads to bind DNA, which also increase their affinity to shorter DNA fragments.

A1. Take Ampure XP beads from fridge to warm to room temperature for 30 minutes.
A2. Prepare buffer containing the following ingredients:

15g PEG-8000;
500μl 1M Tris buffer (pH 8.0);
100μl 0.5M EDTA buffer (pH 8.0);
10ml 5M NaCl;
27.5μl Tween 20;
ddH_2O to fill it to 50 ml.

A3. Take 1 ml Ampure XP beads into an Eppendorf tube. Place the tube onto magnetic rack and wait for 3 minutes until the beads settle down on the wall of the tube. Carefully remove the original buffer. Add 1 ml of the buffer we prepared above. The new beads contain 30% PEG.

B. End preparation

B.1. Take 1/3 of the gDNA solution (the rest is saved to return to the museum and in case that the first trial fails) to a 0.5 ml PCR tube, and add ddH_2O to a volume of 17 μl.
B.2. Add the following solutions.

NEBNext Ultra II End Prep Enzyme Mix (from New England Biolabs, E7645L): 1 μl
NEBNext Ultra II End Prep Reaction Buffer (from New England Biolabs, E7645L): 2.3 μl

B.3. Mix thoroughly. Perform a quick spin to collect all liquid from the sides of the tube.
B.4. Place the tube in a thermocycler, with the lid set to ≥ 75°C, and run the following program:

30 minutes @ 20°C
30 minutes @ 65°C
Hold at 4°C

C. Adapter ligation

C.1. Take 10 μl of NEBNext Adaptor for Illumina (from New England Biolabs, E7335L) into a PCR tube, dilute it by adding 90 μl of ddH_2O.

C.2. Add the following components directly to the End Prep Reaction Mixture:

NEBNext Ultra II Ligation Master Mix (from New England Biolabs, E7645L): 10 μl
NEBNext Ligation Enhancer (from New England Biolabs, E7645L): 0.5 μl
Diluted NEBNext Adaptor for Illumina: 1.2 μl

C.3. Mix thoroughly. Perform a quick spin to collect all liquid from the sides of the tube.

C.4. Incubate at 20°C for 15 minutes in a thermocycler with the heated lid off.

C.5. Add 1 μl of USER™ Enzyme to the ligation mixture.

C.6. Mix well by pipetting up and down. Incubate at 37°C for 15 minutes with the heated lid set to ≥ 45°C.

D. Cleanup the reaction

D.1. Prepare 80% EtOH by mixing 10 ml of ddH_2O and 40 ml pure EtOH.

D.2. Add 3X volume (99 μl) of Ampure XP beads with 30% PEG. Mix by pipetting up and down and allow the beads to bind DNA for 15 minutes.

D.3. Put the tube on magnetic rack for 2 minutes, and remove the supernatant.

D.4. Resuspend the beads using 80% EtOH. Place the tube on the magnetic rack. Wait for 1 minute to let the beads settle down and remove the supernatant.

D.5. Repeat the last step.

D.6. Remove any residual EtOH, dry the beads briefly.

D.7. Add 8 μl DNA elution buffer (ZYMO Research, D3004-4-16) to resuspend the beads. Incubate at 37°C for 2 minutes. Place the beads on the magnetic rack. After the beads settle down, transfer the eluted DNA to a new PCR tube.

D.8. Repeat the last step another time. A total volume of about 15 μl DNA will be obtained.

E. PreCR repair of DNA

E.1. Dilute the 100X NAD solution (from New England Biolabs, M0309L) to 20X with ddH_2O.

E.2. Dilute 100 mM dNTP (New England Biolabs, N0446S) to 2 mM with ddH_2O.

E.3. Add the following reagents to the PCR tube with adapter-ligated DNA from previous step:

10 X ThermoPol Buffer (from New England Biolabs, M0309L): 2μl
20 X NAD: 1μl
2 mM dNTP: 1μl
PreCR repair mix (from New England Biolabs, M0309L): 1μl

E.4. Mix thoroughly. Perform a quick spin to collect all liquid from the sides of the tube.

E.5. Place the tube in a thermocycler, incubate at 37°C for 15 minutes, with the lid set to ≥ 45°C.

F. PCR Eenrichment of adapter-ligated DNA

F.1. Immediately after the PreCR repair, add the following reagents to the PCR tube:

NEBNext Ultra II Q5 Master Mix (from New England Biolabs, E7645L): 25 μl
Index Primer (from New England Biolabs, E7335L): 3 μl
Universal Primer (from New England Biolabs, E7335L): 3 μl

F.2. Mix thoroughly. Perform a quick spin to collect all liquid from the sides of the tube.

F.3. Place the tube on a thermocycler and perform PCR using the following program:

98°C for 30 seconds, 1 cycle
98°C for 10 seconds and then 65 °C for 75 seconds, 12 cycles
65 °C for 5 minutes, 1 cycle
Hold at 4°C

F.4. Measure the concentration of the PCR reaction. If the concentration is >= 2 ng/μl, proceed to the next step. Otherwise, add PCR cycles until the concentration is expected to be about 2 ng/μl.

G. Cleanup the reaction

Note: DNA fragments become longer after adapter ligation and PCR, therefore we are using normal Ampure XP beads to purify the DNA. Because the gDNA fragments are mostly around or below 50bp for historical samples, it is hard to separate them from adapter dimer using beads-based size selection. We will purify both the target DNA and adapter dimers with beads and then use DNA gel for size selection.

G.1. Add 1.8X volume (90 μl) of Ampure XP beads in original buffer (not the beads with 30% PEG). Mix by pipetting up and down and allow the beads to bind DNA for 15 minutes.

G.2. Put the tube on magnetic rack for 2 minutes, and remove the supernatant.

G.3. Resuspend the beads using 80% EtOH. Place the tube on the magnetic rack. Wait for 1 minute to let the beads settle down and remove the supernatant.

G.4. Repeat the last step.

G.5. Remove any residual EtOH, dry the beads briefly.

G.6. Add 20 μl DNA elution buffer (ZYMO Research, D3004-4-16) to resuspend the beads. Incubate at 37 °C for 2 minutes. Place the beads on the magnetic rack. After the beads settle down, transfer the eluted DNA to a new PCR tube.

H. Size selection to remove adapter dimers

H.1. Load the 20 μl eluted DNA into one lane on E-Gel EX Agarose Gels, 2% (Invitrogen G402002). Process 10 samples at a time.

H.2. Add 100bp DNA ladder (New England Biolabs, B7025S).

H.3. Run the gel on E-gel iBase (Invitrogen G6465EU) using preinstalled program for 2% gel in the device.

H.4. Open the gel and visualize the DNA band using E-Gel Safe Imager.

H.5. Identify the adapter dimer band (the first band above 100 bp ladder). Cut the slice the gel containing DNA above 150 bp using a blade. Put the gel slice into a 1.5 ml Eppendorf tube.

I. Recover DNA from gel

I.1. Set the heat block to 50 °C. Make sure EtOH has been added to buffer NT3 (MACHEREY-NAGEL, 740598).

I.2. Weigh an empty Eppendorf tube and the tube with gel slice to determine the weight of the gel (X grams)

I.3. Add 2X ml buffer NT (MACHEREY-NAGEL, 740614.100) and dissolve the gel by incubating the tube at 50 °C. Check every 5 minutes until the gel if fully dissolved.

I.4. Load the dissolved gel onto a Zymo-Spin I column (ZYMO RESEARCH, C1003-50).

I.5. Centrifuge for 1 minute at 10,000g until sample mixtures have been completely filtered. Discard the flow-through.

I.6. Wash the column twice using 500 μl (each time) buffer NT3 (MACHEREY-NAGEL, 740598).

I.6. Centrifuge for 2 minutes at 10,000g to dry the column.

I.7. Add 20 μl DNA elution buffer (ZYMO Research, D3004-4-16) and incubate at 50 °C for 2 minutes.

I.8. Spin for 2 minutes at 10,000g to collect the eluted DNA into an Eppendorf LoBind tube.

J. Dissect genitalia

Note: if the library preparation is successful, we will proceed to genitalia dissection which will ruin all the remaining DNA in insect abdomen. If the library is not successful, we will try to extract gDNA from the abdomen another time.

J.1. Set the heat block to 60°C.

J.2. Soak the abdomen for 40 minutes in 10% KOH at 60°.

J.3. Operating under dissecting microscope, clean the genitalia from remaining scales to expose the genitalia.

J.4. Store the dissected genitalia in a small glycerol-filled vial and label it.

I.3. Reference genome assembly and annotation

We sequenced and assembled a reference genome from a *Hesperia colorado sublima* (ADW0057) specimen as previously described (Cong, et al. 2015a, b). Briefly, paired-end libraries with insert sizes 250 bp and 500 bp and mate-pair libraries with insert sizes 2 kb, 5 kb, and 10 kb were constructed and sequenced using Illumina platform. To facilitate genome annotation, we also prepared RNA-seq library from another *Hesperia colorado sublima* specimen (ADW0056). All the reads were processed by Trimmomatic (Bolger, et al. 2014) to remove adapter sequences and low-quality (quality score < 20) bases, and genomic DNA reads were further processed by Quake (Kelley, et al. 2010) to correct sequencing errors. We used Platanus (Kajitani, et al. 2014) to assemble the genomes. The initial assembly from Platanus was redundant: the highly heterozygous equivalent segments in the paternal and maternal chromosomes were treated separately, and thus they were present twice in the assemblies. We detected and corrected such problems as described before (Cong, et al. 2015a, b).

We used RepeatModeler <http://www.repeatmasker.org/RepeatModeler/> to identify repeats in the genome. In addition, since repeats with highly similar sequences may be erroneously combined into one in the genome assembly, we identified them using very high sequence depth (more than 4 times of the expected value) after mapping all the sequence reads to the draft genome using BWA (Li and Durbin 2009). We combined the repeats identified by RepeatModeler and our sequence depth criteria with repeats in Repbase (Bao, et al. 2015) to generate species-specific repeat libraries, and these libraries were supplied to RepeatMasker <http://www.repeatmasker.org/> to annotate repeats in the genome.

We annotated protein-coding genes in the genome using three approaches: homology-based, transcript-based, and *de novo* gene prediction. We used protein sets from *Papilio machaon* (Li, et al. 2015), *Pieris rapae* (Shen, et al. 2016), *Calycopis cecrops* (Cong, et al. 2016), *Calephelis nemesis* (Cong, et al. 2017), *Danaus plexippus* (Zhan, et al. 2011), *Cecropterus lyciades* (Shen, et al. 2017), *Bombyx mori* (Kawamoto, et al. 2019), and *Drosophila melanogaster* (dos Santos, et al. 2015) as references for homology-based annotation. The reference protein sets were aligned to the genome assembly using exonerate (Slater and Birney 2005). We aligned the RNA-seq reads to the reference genome using TopHat (Trapnell, et al. 2009), and derived transcript-based annotations using Cufflinks (Trapnell, et al. 2010). Three *de novo* gene prediction methods: Augustus (Stanke, et al. 2004), GeneMark_ES (Lomsadze, et al. 2005), and SNAP (Korf 2004) were used to obtain *de novo* gene annotations. We trained these *de novo* gene predictors for the *Hesperia colorado* genome using confident gene models derived from the consensus between transcript-based and homology-based annotations. Finally, annotations by different approaches were combined in EvidenceModeler (Haas, et al. 2008) to obtain their consensus as the final gene predictions. We predicted

the functions of these proteins by finding the closest homologs in Flybase (Thurmond, et al. 2019) and Swissprot (UniProt Consortium 2019) using BLASTP (Altschul, et al. 1990) (E-value < 0.00001) and transferred the Gene Ontology (GO) (Gene Ontology Consortium 2015) terms and function annotations.

High conservation of gene content has been reported in Lepidoptera Z chromosome (Fraisse, et al. 2017), and therefore we can deduce the Z-linked genes in other species by comparing to *Heliconius melpomene* reference genome, where Z chromosome sequence was known (Heliconius Genome Consortium 2012). We split all *Heliconius melpomene* proteins into exons and searched for the closest hit (sequence identity > 60%) of each exon in the *Hesperia colorado* reference genome. We calculated the fraction of Z-linked exons that are mapped to each scaffold out of all exons, and if this fraction is larger than 75%, we consider this scaffold to be putative Z-chromosome.

We also assembled and annotated the reference mitogenome of *Hesperia colorado* using specimen LEP28439, which is fresh and contains a high fraction of mitochondrial reads. Using the mitogenome of *Lerema accius* (accession: NC_029826.1) (Cong and Grishin 2016) as reference, we identified the reads that can be mapped to it using BWA (Li and Durbin 2009). We assembled these reads into scaffolds *de novo* using Platanus (Kajitani, et al. 2014). The scaffolds were manually inspected to remove redundant sequences and merge overlapping ones, resulting in a nearly complete mitogenome except the D-loop region. We manually completed the sequence in that region by extending the sequence with reads that overlap with the termini of the sequence by at least 40 bp until some reads can connect the 3’ end with the 5’ end, and complete the circular DNA. We annotated the mitogenome using mitochondrial protein sequences from *Lerema accius.*

I.4. Assembling genomic sequences

We assembled the genomes of other specimens by mapping the reads to the reference and SNP calling. We removed the sequencing adapters and low-quality portions from sequencing reads using Trimmomatic (Bolger, et al. 2014), and merged read1 and read2 from the same fragment if their sequences overlap using PEAR (Zhang, et al. 2014). The resulting reads of each specimen were mapped to the reference genome using BWA (Li and Durbin 2009). We kept the reads that are mapped unambiguously in the correct orientation from the BWA’s result. Using the BWA results, we computed the total sequencing depth for all recently collected specimens (after 2015) in each 100 bp window. We considered windows with too high or too low total depth to be less confident. For example, windows with high depth might cover repetitive regions and might not be suitable for phylogenetic studies, whereas windows with very low depth may be misassembled or highly variable. Therefore, we only used the windows with depth in between half of the median and twice the median sequencing depth, and reads mapped to genomic regions other than these windows were discarded. Since many specimens were historical and their DNA could be contaminated, we developed and sequentially applied the following two protocols to clean up the alignments.

Protocol A. For each 30 bp sliding window applied to the alignment between the reference genome and the reads, we clustered all the reads into groups of similar sequences using the following procedure. We ranked reads based on their sequence identity to the query from high to low. The first read initiated a

cluster. Starting from the second read, each new read was compared to the first sequence of each cluster and assigned to the first cluster whose first sequence had no more than one mismatch from the current sequence. If a new read could not be assigned to existing clusters, a new cluster was initiated with this read as the first member. For each cluster, we computed its size and the average number of mismatches to the query and we considered a cluster to be good if its size was at least half of the largest cluster size and the number of mismatches was no larger than the minimal mismatches for all clusters + 2. If the number of good clusters was no more than 2, we marked the reads that were not in the good clusters as bad reads; otherwise, we marked all the reads as bad. All the bad reads were discarded.

Protocol B. For each read (a target), we got other reads that overlapped with it in the genomic regions they mapped to and counted the number of reads that were consistent and inconsistent with it. We considered a read to be consistent if it overlapped with the target read for at least 20 bp and the sequence identity was above 95%; otherwise, we considered a read to be inconsistent if it had at least 2 mismatches from the target and sequence identify <95 %. If the number of consistent reads for a target was more than twice the number of inconsistent reads, the target read was kept. Otherwise the target read was discarded.

We performed SNP calling for each specimen using samtools (Li, et al. 2009) and the alignments between reference and reads after the two cleaning-up protocols. For PCA (Price, et al. 2006), t-SNE (van der Maaten and Hinton 2008), and STRUCTURE (Pritchard, et al. 2000) analyses, we derived the genomic sequence of each specimen by taking the SNPs called at positions that are covered by at least two different reads and filling the remaining positions with gaps. To prepare the input for TreeMix (Pickrell and Pritchard 2012), we did not perform SNP calling. Instead, we recorded the frequency of each nucleotide in the sequencing reads at each position. To prepare the input for phylogenetic reconstruction, we obtained the dominant (frequency > 0.6) nucleotide at each position. A very similar protocol was used to obtain the mitochondrial genomes. Since mitogenome is haploid, we modified protocol A to allow only one good cluster, and we took the dominant nucleotide at each position for each specimen to determine its sequence.

Section II: Population Genetic study of *Hesperia colorado* populations in Colorado State of the United States.

II.1. Handling specimens with poor data quality

A unique challenge of this study is to properly analyze the historical museum specimens with poor data quality. A leg of a specimen of 150 years old contains a low amount (less than 0.1 ng) of genomic DNA. Genomic DNA from these specimens were typically fragmented to about 50 bp in size, and thus we could only obtain about 50 bp useful sequence (and the rest will be adapter sequences or random sequences after the adapter is completely sequenced) from one fragment even if we sequenced 150 bp at both ends (300 bp total data). Therefore, only about 1/6 of the sequencing data from an historical specimen was retained after processing by Trimmomatic and PEAR. In addition, the historical specimens contain more contaminations, and thus a large fraction of data were removed in our cleaning-up procedure. As a result,

assembled genomes for historical specimens were highly incomplete (Table S4). Out of the 86 *Hesperia colorado* specimens, 14 contain more than 50% gaps (NVG-5304, NVG-5533, NVG-6313, NVG-6705, NVG-6706, NVG-6708, NVG-7574, NVG-7575, NVG-15111B01, NVG-15111B02, NVG-16108A06, NVG-16108A07, NVG-16108A08, and NVG-16108C04). Majority of these specimens are between 50 and 150 years old, while we did not obtain sufficient sequence reads for other specimens because the amount of data we got was less than we planned and it could have been compensated by additional sequencing of these specimens if needed.

Population genetic tools such as Eigensoft (Price, et al. 2006) and TREEMIX (Pickrell and Pritchard 2012) are based on covariation of allele frequencies between specimens. Since the covariances are computed on the shared positions between specimens, and calculation for each pair of specimens will be done on very different sets of positions if there are a high fraction of gaps in some specimens (Fig. S1). In addition, we observe that variable regions in the genome tend to have more gaps than conserved segments, and this trend is significantly more obvious in historical specimens (Fig. S2). Therefore, regions that are missing (gaps) in historical specimens are on average more divergent than the regions that are present. If we include a lot of segments that are missing in the historical specimens, we will underestimate the divergence between historical specimens and other specimens because of the shortage of more divergent regions. These two factors imply that ideally, these analyses should be done on sequence alignments with minimal amount of gaps in each specimen.

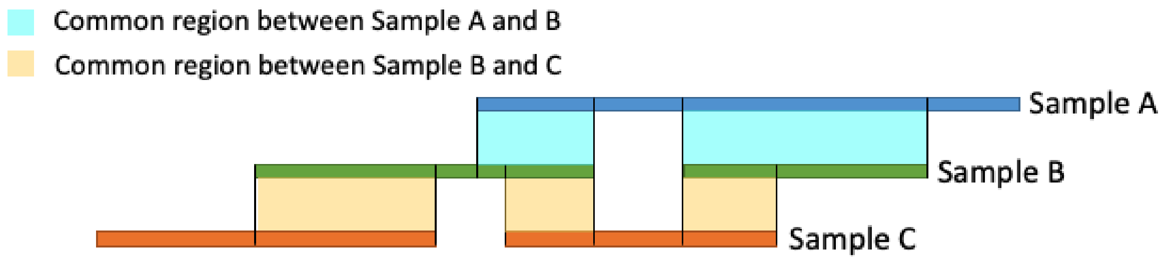

Fig. S1. Having a large fraction of gaps in the sequence alignment can bias covariance calculations. The covariance between specimen A and B will be based on the regions highlighted in cyan and the covariance between specimen B and C will be based on the regions highlighted in yellow orange.

However, removal of positions with gaps drastically decreases the amount of data that can be used. We therefore need to balance between removing positions with gaps and retaining a sufficient amount of positions in the alignment to reflect the evolutionary history. Since gaps in the sequence alignment of all *Hesperia colorado* specimens are mostly introduced by the historical and poorly covered specimens. One way to solve this dilemma is to do the analyses first without the 14 poorly covered specimens. We applied several population genetic tools on the 72 well covered specimens to identify the specimens showing hybrid origins (detailed below), and the hybrids we identified include LEP-28422, LEP-28426, NVG-5300, NVG-5532, NVG-16108A01, NVG-16108A02, NVG-16108C10, and NVG-16108C11. The remaining 64 specimens were used as “backbone” specimens to place the 14 poorly covered specimens.

We term these 14 specimens “targets” to place into the population structure defined by the “backbone” specimens. We used only one of the 14 targets at each time, and performed the population genetic

analyses with all the backbone specimens and one target specimen. We then integrated results from these analyses with slightly different sets of specimens (backbone plus one target). The integration methods are different for each software, and the details are described in the following subsections.

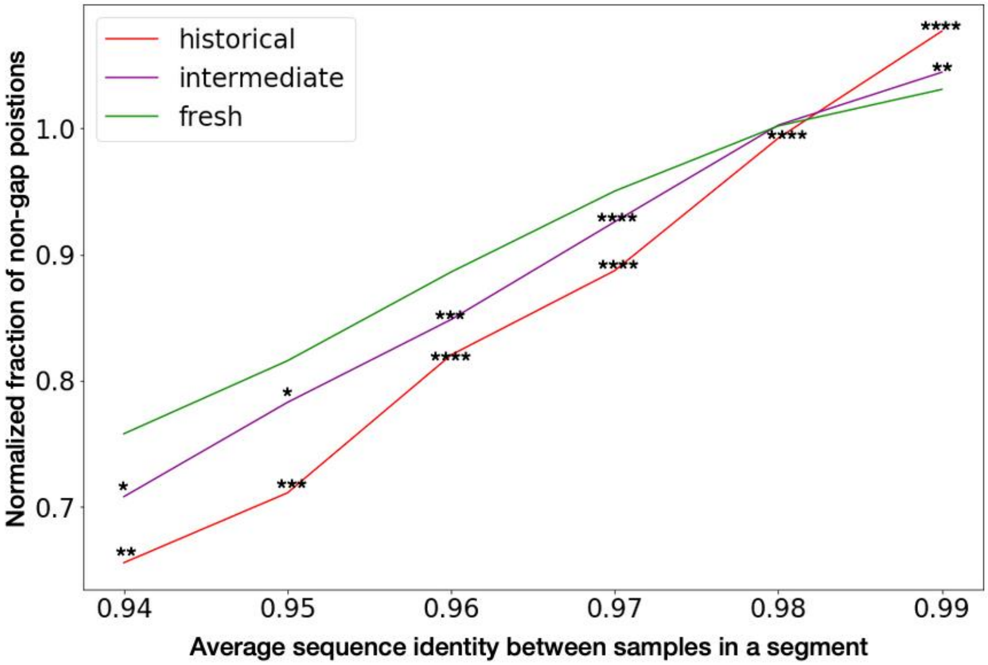

Fig. S2. Variable regions in the genome are poorly covered in historical specimens. The genome is partitioned into 100 bp segments and the average sequence identity between *H. colorado* specimens in CO state is computed to indicate the level of divergence of that segment. We partitioned the samples by their age to three groups: historical samples (> 100 years old), fresh samples (freshly preserved in *RNAlater* or alcohol), and intermediate samples (30-50 years old). We computed the average fraction of non-gap positions in each specimen in segments belonging to each "sequence identity bin" and normalized that value by the average fraction of non-gap positions over the entire genome for that specimen. The normalized non-gap ratio for specimens in a group was averaged for each bin and each group. We compared the average non-gap fraction of historical or intermediate specimens to the fraction for fresh specimens in each "sequence identity bin", and the significance level for the difference between historical or intermediate specimens and fresh specimens are evaluated using student's T-test.

II.2. Alignment processing

To prepare the input files for PCA and STRUCTURE, we processed the alignment with all the 72 well-covered specimens (discussed above) to remove positions with gap ratios above a certain cutoff: four gap ratio cutoffs were used here: 0.1, 0.15, 0.2, and 0.25. Ideally, we would like each specimen to contain a similar fraction of gaps. If we directly remove positions where the fraction of gaps is above the cutoff, the numbers of gaps in different specimens after removal will be highly uneven. We invented a trick to solve this problem by partitioning the specimens into 3 groups according to the overall gap fraction in each specimen. Specimens with gap fraction between 0.35 and 0.5 (group1), between 0.2 and 0.35 (group2), and between 0 and 0.2 (group3) are partitioned into three groups, respectively. Starting from the group with the highest gap fraction, we computed the fraction of specimens in this group that are gap in each

position, and removed positions with gaps more than the cutoff (0.1, 0.15, 0.2, or 0.25). Three rounds of gap removal were sequentially performed, using specimens in group1, group2, and group3, respectively.

We processed the alignment of 64 “backbone specimens” similarly. In order to place the “target” specimens, we add one “target” to the alignment of “backbone” specimens at each time. Upon addition of a specimen, we kept all the positions that were not gap in this additional specimen, and randomly selected some positions that were gap in the additional specimen to ensure that the gap fraction in the additional specimen matched the gap ratio cutoff (0.1, 0.15, 0.2, or 0.25).

We next selected confident biallelic loci from the alignment after gap filtering (either an alignment with all well-covered specimens or the alignment with one “target” and all “backbone” specimens). We considered positions with two possible nucleotides, and required each nucleotide to show up in at least three specimens. We ignored low frequency SNPs present in less than 3 specimens because they may represent errors in sequencing. We ignored positions with more than two possible nucleotides. Such positions are rare because all the specimens are closely related, and the number of triallelic loci is about 20 times less than biallelic loci.

We further selected representative positions among linked loci using plink (Purcell, et al. 2007) <http://pngu.mgh.harvard.edu/purcell/plink/>. We converted the alignment into the input files (*.ped and *.map) of plink according the online manual <http://zzz.bwh.harvard.edu/plink/data.shtml> and we ran plink with the following commands: plink --file [input basename] --noweb --indep-pairwise 50 10 0.1; plink --file [input basename] --noweb --extract plink.prune.in --make-bed --out [output basename], where “plink.prune.in” is the output from the first command.

II.3. Decomposing genomic data with Principal Component Analysis (PCA) and clustering the specimens with t-SNE

We counted the number of positions after processing under different gap ratio cutoffs, and we selected the cutoff resulting in 50,000 - 100,000 positions for each input alignment. If more than one cutoff produced alignments with positions in this range, we chose the lower cutoff (fewer gaps allowed). A cutoff of 0.15 was selected for most cases, and a cutoff of 0.2 or 0.25 was used for some alignments with the oldest specimens. The processed alignments were converted to inputs (*.snp, *.geno, and [*.ind) according to the user manual <https://github.com/chrchang/eigensoft/tree/master/CONVERTF>. In addition, we prepared a parameter file (below is an example) for each dataset. Finally, we ran Eigensoft by: smartpca -p [parameter file], where smartpca is the program for principal component analysis from the Eigensoft package.

```
genotypename: [input basename].geno
snpname: [input basename].snp
indivname: [input basename].ind
evecoutname: [output basename].evec
evaloutname: [output basename].eval
altnormstyle: NO
numoutevec: 10
```

numoutlieriter: 5
numoutlierevec: 10
outliersigmathresh: 6
qtmode: 0

We first obtained the PCA result for the 72 well-covered specimens. We visualized the first two principal components using python matplotlib <https://matplotlib.org/> and obtained Fig. S3. This analysis partitioned the specimens into four groups as indicated by the color of dots in Fig. S3: *Hesperia colorado sublima* (red), *H. c. ochracea* (orange), *H. c. colorado* (blue), and *H. c. idaho* (green). There are specimens in between these populations (black dots), and they are possible hybrids of different populations. The hybrid origin of these specimens is also supported by STRUCTURE. The distinct populations (subspecies) and hybrids revealed in our analysis are in agreement with the geographical location and phenotypes of these specimens: *sublima* corresponds to the population on the mountain top, and the other three populations live in lower elevation, separated from each other by mountain chains.

We excluded the hybrid specimens (black dots in Fig. S3) and performed another PCA with all the backbone specimens (Fig. S4). Similarly, we performed PCA for each target specimen with the backbone specimens and the results are in Figs. S5-S7. These analyses placed the targets into different populations. In consistency with the phenotype, NVG-5533, NVG-16108A06, NVG-15111B01, NVG-15111B02, and NVG-6705 are grouped with *H. c. ochracea* (the orange dots) by PCA. The collecting localities of NVG-5533, NVG-16108A06 and NVG-6705 also suggest that they belong to *H. c. ochrecea*. NVG-15111B01 and NVG-15111B02 are type specimens of *H. c. ochrecea*, but the exact localities for them are not known. NVG-7574, NVG-7575, NVG-16108A07, NVG-16108C04, NVG-6313, and NVG-6708 are grouped with *H. c. colorado* (the blue dots). The first four specimens were collected in the range occupied by *H. c. colorado*, and thus the genomic data agree with the geographic records of them. NVG-6313 and NVG-6708 are the lectotype and paralectocype of *H. c. colorado*, respectively. Their collecting locality was suggested to be Lake County based on the diary of the collector and the labels (collecting date) on the specimens. NVG-16108A08 grouped with *H. c. idaho* specimens, which is also consistent with its collecting locality on the label of the specimen. Two specimens appear to have hybrid origins: NVG-6706 and NVG-5304. Both of them were collected in areas near the high elevation population, *H. c. sublima*, and thus it is reasonable for them to contain a significant portion of genes from *H. c. sublima*. NVG-6705 and NVG-6706 are expected to be as old as the lectotype (NVG-6313) and paralectotype (NVG-6708) of *H. c. colorado*, and correctly grouping them with the expected populations suggests that our analytical methods work for specimens of this age.

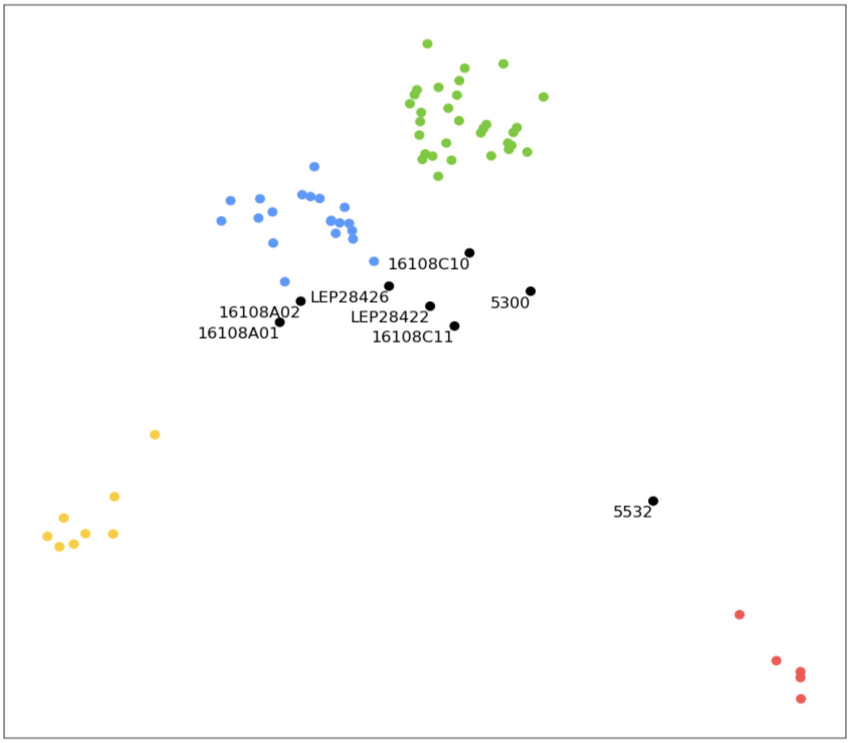

Fig. S3. The first two principal components for the genetic variations in the 72 well-covered specimens. *H. c. sublima*, *H. c. ochrecea*, *H. c. colorado* and *H. c. idaho* specimens are colored in red, orange, blue, and green, respectively. Specimens of hybrid origin revealed by STRUCTURE are colored in black and labeled by the specimen IDs. These specimens appear to be in between populations in PCA as well.

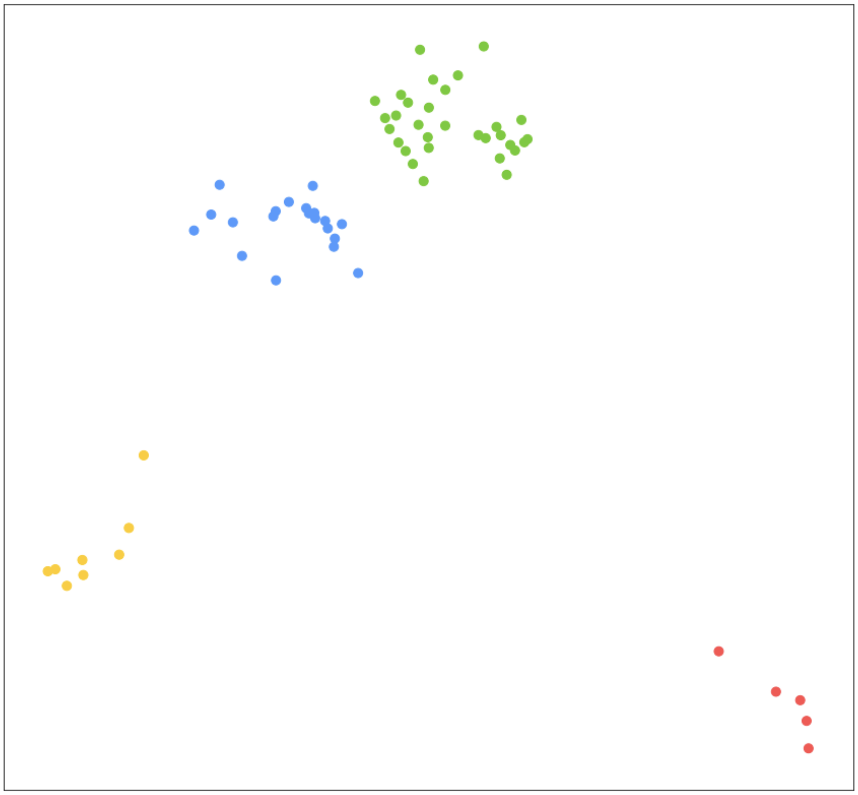

Fig. S4. The first two principal components for the genetic variations in the 64 backbone specimens. The color scheme is the same as Fig. S3. All other PCA results are superimposed onto this one to generate Fig. 2a that includes all specimens.

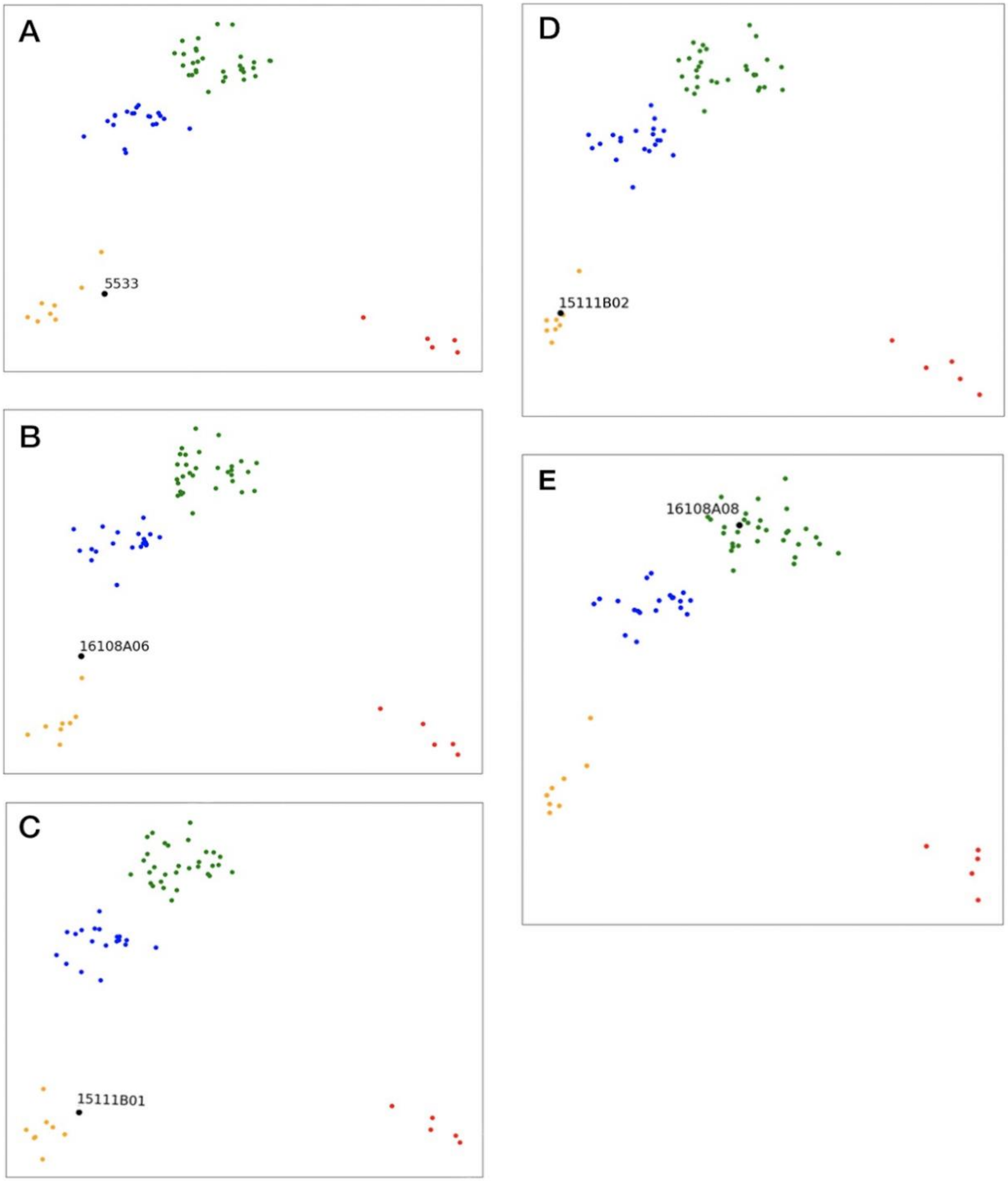

Fig. S5. The first two principal components for the genetic variations in specimens (A) NVG-5533, (B) NVG-16108A06, (C) NVG-15111B01, (D) NVG-15111B02, and (E) NVG-16108A08 with backbone specimens representing the four subspecies (populations). The color scheme is the same as Fig. S3.

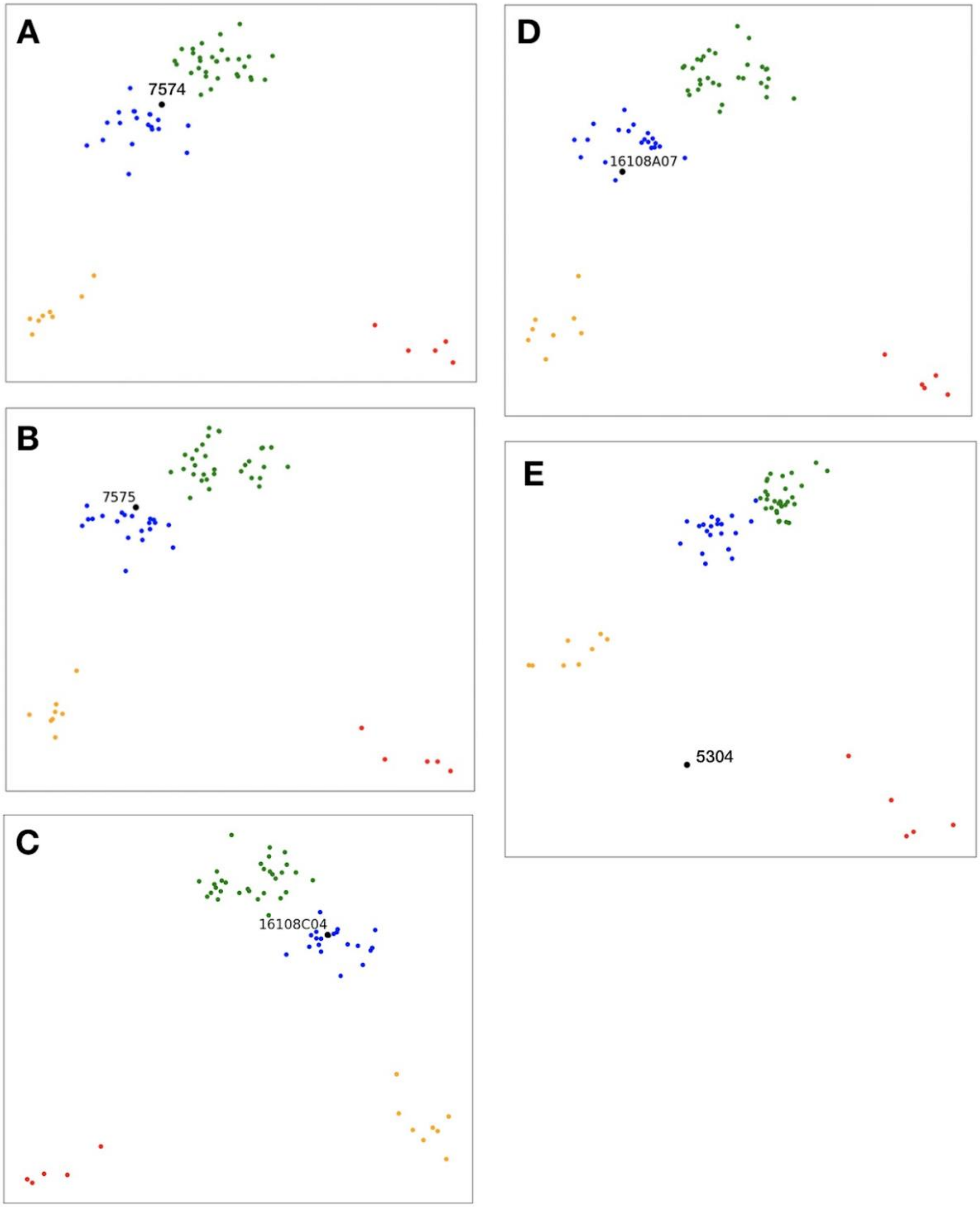

Fig. S6. The first two principal components for the genetic variations in specimens (A) NVG-7574, (B) NVG-7575, (C) NVG-16108C04, (D) NVG-16108A07, and (E) NVG-5304 with the backbone specimens representing the four subspecies (populations). The color scheme is the same as Fig. S3.

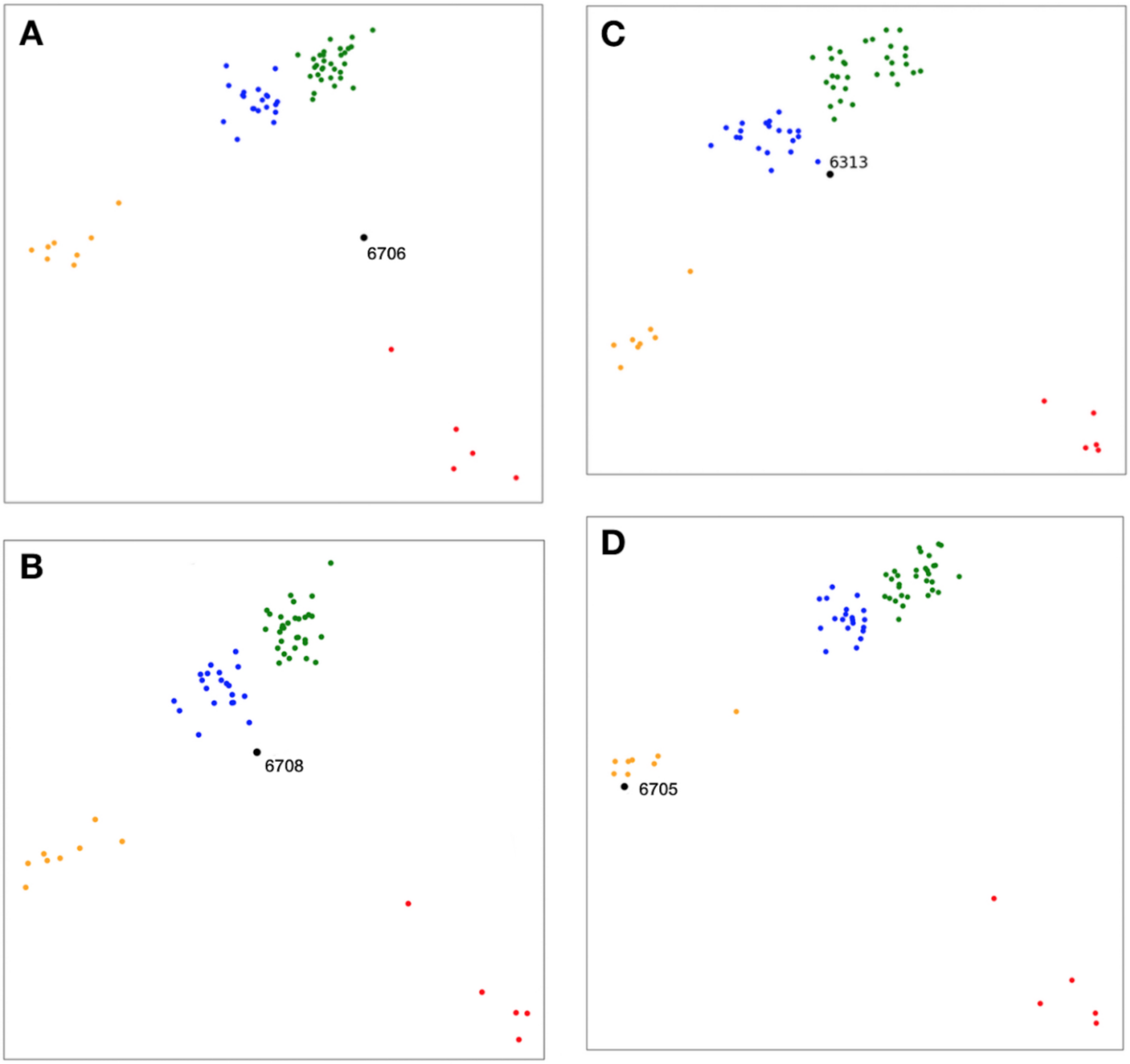

Fig. S7. The first two principal components for the genetic variations in specimens (A) NVG-6706, (B) NVG-6708, (C) NVG-6313, (D) NVG-6705 with backbone specimens representing the four subspecies. These specimens are all about 150 years old. NVG-6313 is the lectotype of *Hesperia colorado colorado* (the blue dots), and NVG-6708 is likely a paralectotype collected together with NVG-6313. The color scheme is the same as Fig. S3.

In order to visualize all the target specimens together with the backbone specimens, we merged the visualization of PCA done for each specimen to the one performed on all well-covered specimens. We considered each of the PCA results displayed in Figs. S3-S7 as a 2D image and we wanted to align all the images in Fig. S3, S5A-E, S6A-D, and S7A-D to the one in Fig. S4 (PCA with backbone specimens). We find the transformations that make the coordinates of backbone specimens (they are present in all the images) superimposed between different images with the minimal root mean square distances. We allow

translation, rotation, and rescaling in these transformations, and the python script to find the best transformation is outlined below.

```
import numpy, math, scipy
import scipy.optimize
from scipy.optimize import minimize
# original_dots is a dictionary with the coordinate for each specimen in the PCA result to be transformed (Fig. S3 and each panel in Figs. S5-S7).
# target_dots is a dictionary with the coordinate for each specimen in the PCA result of backbone specimens only (Fig. S4)
# common_names are the names for backbone specimens shared among all PCA results
# unique_names are the unique names in each of the PCA results, i.e., hybrid specimens in Fig. S3, and the “target” specimens in panels from Figs. S5-S7.

def transform(x,y,scalx,scaly,tranx,trany,alpha):
    rot_mtx=numpy.array([[math.cos(alpha),-math.sin(alpha)],[math.sin(alpha),math.cos(alpha)]])
    trans_mtx = numpy.array([scalx*(x-tranx),scaly*(y-trany)])
    return numpy.matmul(rot_mtx,trans_mtx)
def get_sd(params):
    alpha = params[0]
    scalx = params[1]
    scaly = params[2]
    tranx = params[3]
    trany = params[4]
    dist = 0
    for name in common_names:
        ox = original_dots[name][0]
        oy = original_dots[name][1]
        tx = target_dots[name][0]
        ty = target_dots[name][1]
        [nx, ny] = transform(ox,oy,scalx,scaly,tranx,trany,alpha)
        dist += (nx - tx) ** 2 + (ny - ty) ** 2
    return dist ** 0.5
start_params = [0,1,1,0,0]
best_params = minimize(get_sd, start_params, method='L-BFGS-B', tol=1e-10)
```

The transformations found with the above script were applied to the coordinates of the unique specimens in each PCA, and the script to perform the transformation is:

```
[alpha,scalx,scaly,tranx,trany] = best_params.x
for name in unique_names:
    orig_x = need_dots[name][0]
    orig_y = need_dots[name][1]
    [tran_x, tran_y] = transform(orig_x,orig_y,scalx,scaly,tranx,trany,alpha)
```

The transformation allows the coordinates for the first two principal components in different analyses to superimpose onto each other with minimal RMSD, and the transformed coordinates of the hybrids and

target specimens are placed in the coordinates for PCA result with the backbone specimens only in Fig. S4 to obtain Fig. 2a (main text).

Eigensoft by default outputs 10 principal components, and the eigenvalues of these components for the analysis on all 72 well-covered specimens are shown in Fig. S8. We do not see a drastic decrease in the Eigenvalues for principal components after the first two, and this is true for all other PCAs done for different sets of specimens. In order to capture all the signal in different principal components, we summarized all the 10 principal components using t-SNE, a tool to cluster data points in high-dimensional space and reduce the dimension (van der Maaten and Hinton 2008). T-SNE does not work well with hybrids, because they cannot be clustered into any group, thus we did not apply it to PCA results containing hybrids. In addition, sometimes the principal components reflect the difference in data quality. For example, the 3rd principal components in the analysis of NVG-6705 or NVG-6708 with all backbone specimens places NVG-6705 or NVG-6708 far away from the rest, possibly due to higher levels of degradation and contamination (some contamination remains after the cleaning up routines) for these specimens over 150 years old. Therefore, for these two specimens, we only used the first two principal components to avoid biases from the data quality issues.

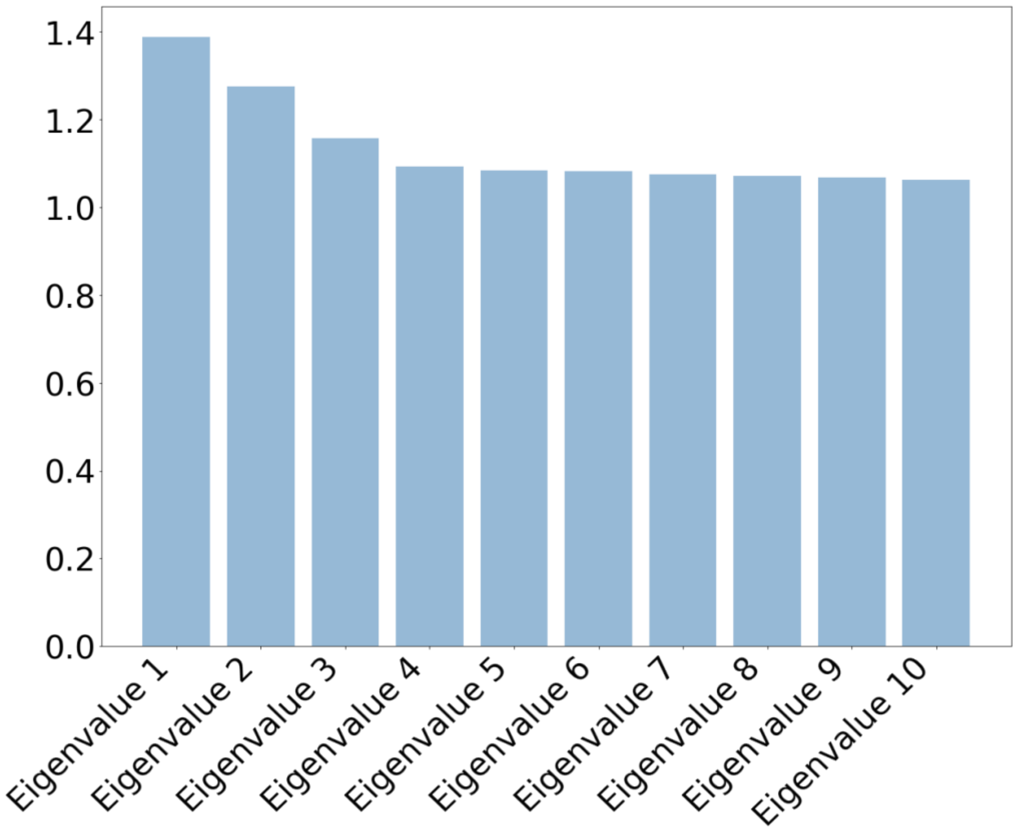

Fig. S8. Eigenvalues for the principal components of the genetic variations in the 72 well-covered specimens (Fig. S3).

We performed t-SNE using the following commands: tsne = TSNE(n_components=2, random_state=100, perplexity=7, n_iter=20000); dots_2d = tsne.fit_transform(dots), where dots_2d is the output array with the vector for each specimen after t-SNE transformation, and dots is the input array with the vector for each specimen. T-SNE needs an important parameter, perplexity, to indicate the expected size of each cluster. The number of specimens in each subspecies is drastically different in our case, from 5 to 29, and we find that perplexity of 6 or 7 usually gives results that are consistent with geography, the first two principal components and STRUCTURE. T-SNE results for the backbone specimens only and the backbone specimens with each target specimen are shown in Figs. S9-S11. T-SNE partitioned the specimens into 4 major clusters. Since the expected size of each cluster we set is 6 or 7, the subspecies containing more specimens, *H. c. colorado* and *H. c. idaho* can be partitioned into smaller minor clusters.

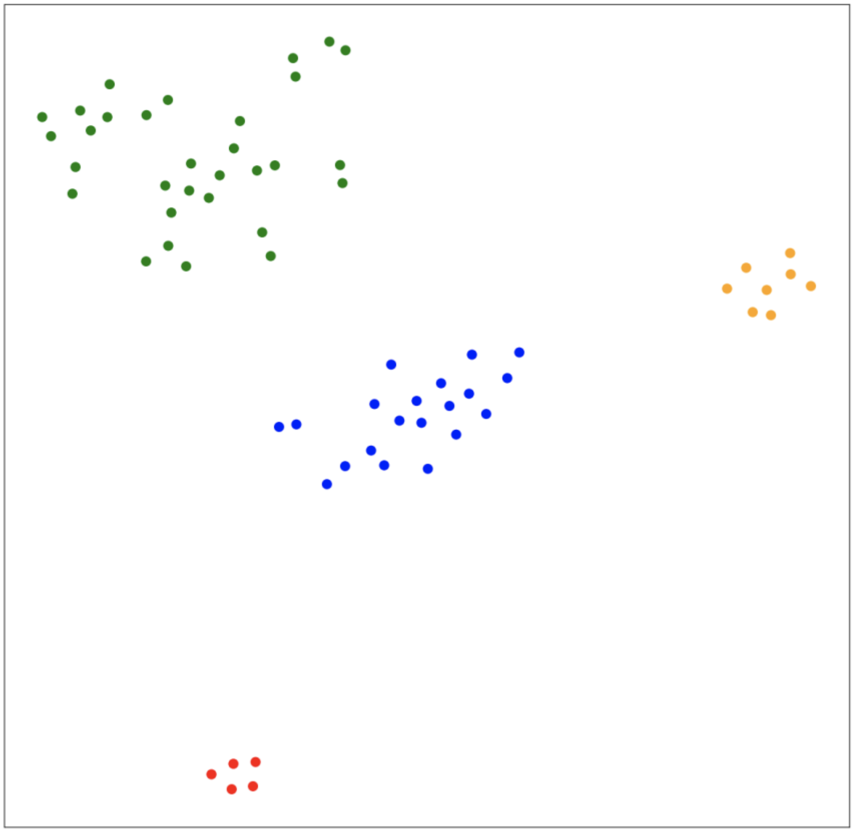

Fig. S9. Clustering of non-hybrid backbone specimens with t-SNE using the first 10 principal components of the genetic variations. The color scheme is the same as Fig. S3.

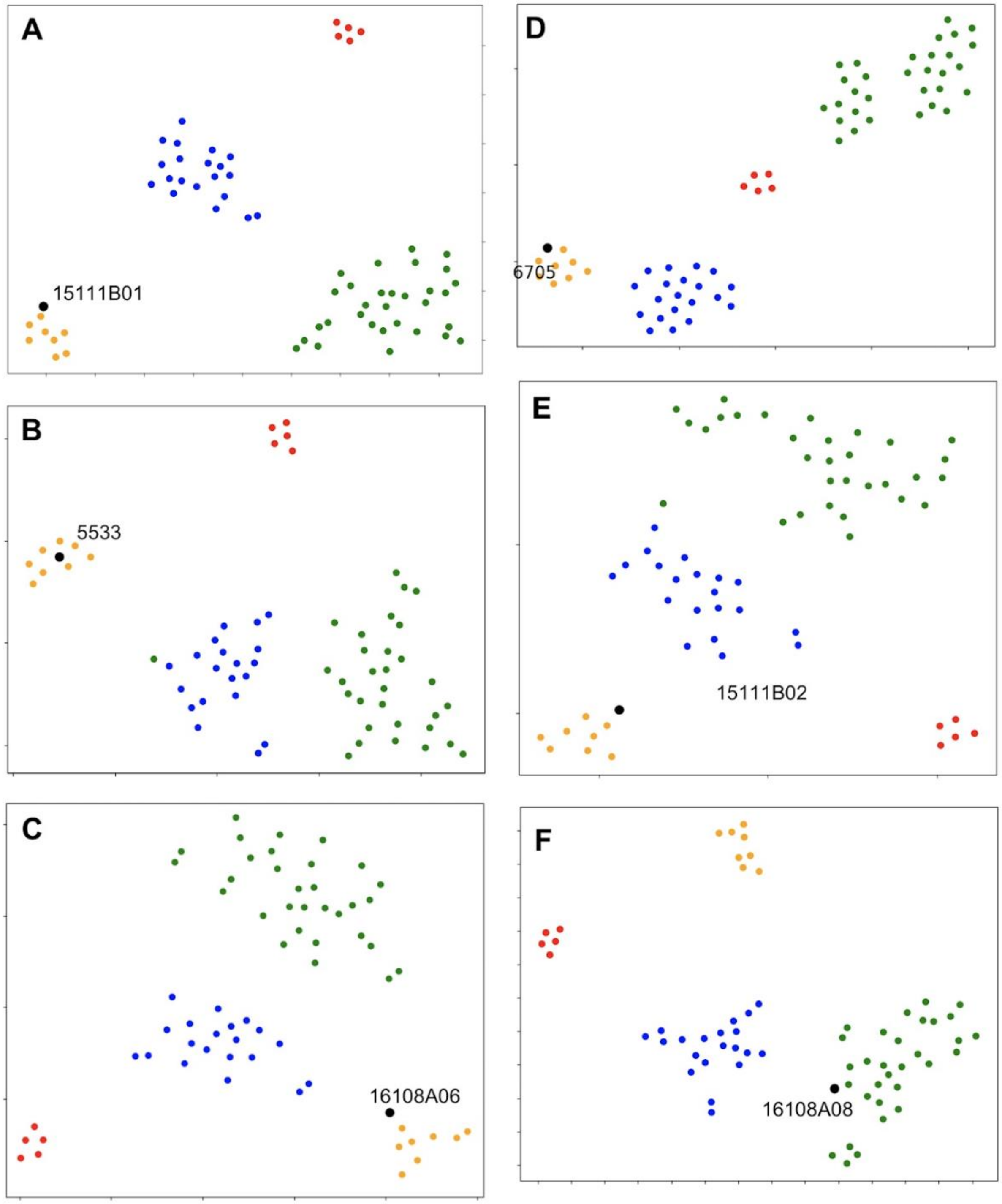

Fig. S10. T-SNE results for (A) NVG-15111B01, (B) NVG-5533, (C) NVG-16108A06, (D) NVG-6705, (E) NVG-15111B02, and (F) NVG-16108A08 together with the backbone specimens. The color scheme is the same as Fig. S3. The black dot represents the target specimen that is being placed in the framework of the backbone specimens.

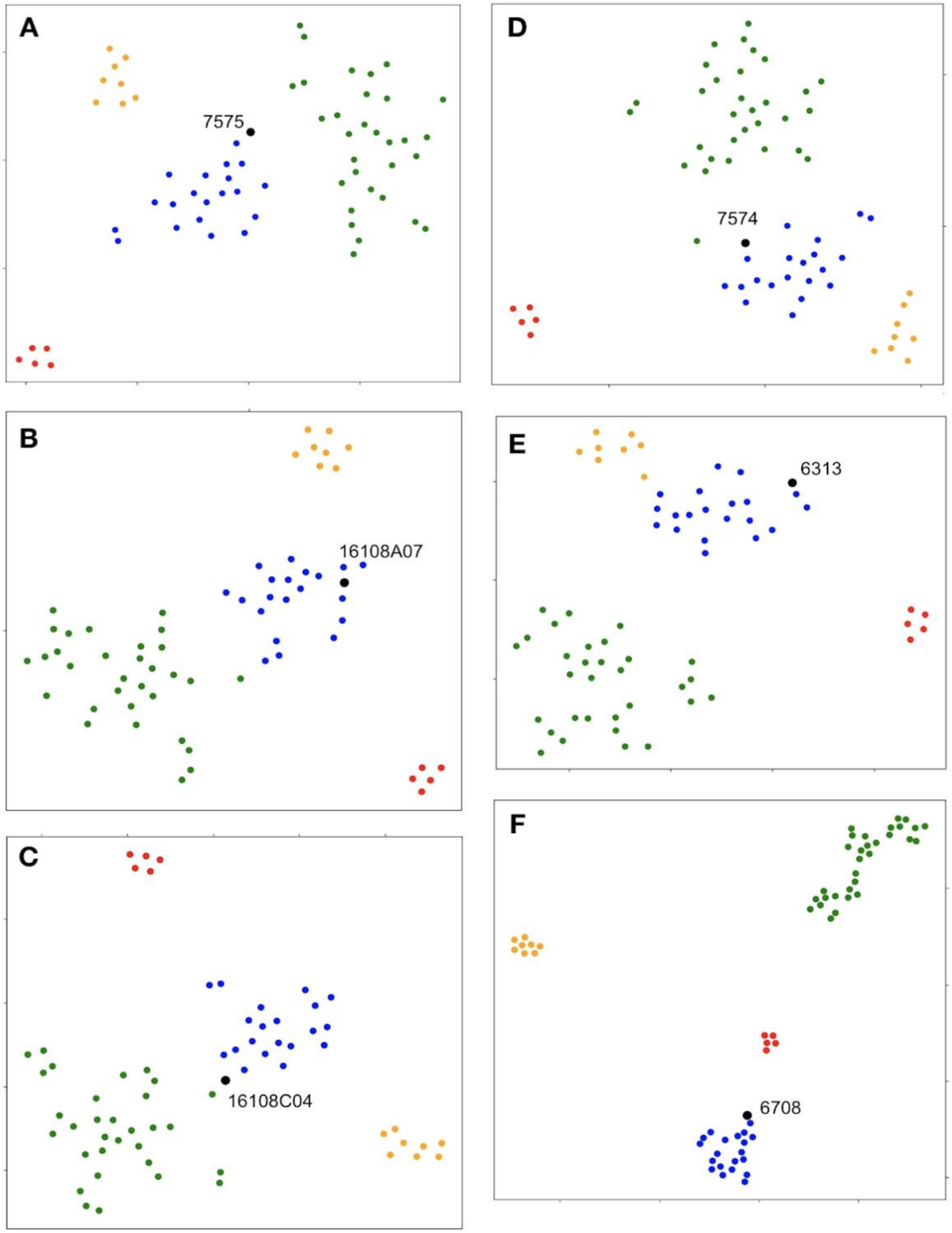

Fig. S11. T-SNE results for (A) NVG-7575, (B) NVG-16108A07, (C) NVG-16108C04, (D) NVG-7574, (E) NVG-6313, (F) NVG-6708 together with the backbone specimens. The color scheme is the same as Fig. S3. The black dot represents the target specimen that is being placed in the framework of the backbone specimens.

To visualize the t-SNE results for all the targets together, we merged the panels in Figs. S10 and S11 to Fig. S9 by placing the additional specimen (target specimen) into the coordinates of backbone specimens in Fig. S9. For each target, we extracted 5 specimens from each subspecies with the smallest distance to it in the t-SNE analysis done on this specimen together with the backbone specimens, and we summarized the distances from these 5 specimens as the distance from the target specimen to a subspecies. For a target species, its distance from the 4 subspecies are thus represented as a vector, [dist1, dist2, dist3, dist4]. We normalized this vector using the smallest distance among dist1, dist2, dist3, and dist4 to obtain normalized_distance_vector = [dist1/mindist, dist2/mindist, dist3/mindist, dist4/mindist]. When placing the target specimen in the backbone-only t-SNE result (Fig. S9), we calculated the distances between target specimen and the same sets of specimens from each subspecies and obtained another vector [dist1', dist2', dist3', dist4'], which was normalized similarly to obtain normalized_distance_vector' = [dist1'/mindist', dist2'/mindist', dist3'/mindist', dist4'/mindist']. We searched for the coordinate for each target specimen to minimize the difference between normalized_distance_vector and normalized_distance_vector'. The python script to carry out the above calculations is shown below. The coordinates for target specimens were used to display them together with the backbone specimens in Fig. 2c of the main text.

```
import scipy
import scipy.optimize
from scipy.optimize import minimize

def get_dist(Adot, Bdots):
    dists = []
    for Bdot in Bdots:
        dist = ((Adot[1] - Bdot[1]) ** 2 + (Adot[2] - Bdot[2]) ** 2) ** 0.5
        dists.append([Bdot[0],dist])
    dists.sort(key=lambda x:x[1])
    total_dist = 0
    ref_dots = []
    for dist in dists[:5]:
        ref_dots.append(dist[0])
        total_dist += dist[1]
    return [ref_dots, total_dist]

def get_residual(new_coor, set1, set2, set3, set4):
    dist1 = 0
    for name in set1:
        ref_coor = ref_coors[name]
        dist1 += ((new_coor[0] - ref_coor[0]) ** 2 + (new_coor[1] - ref_coor[1]) ** 2) ** 0.5
    dist2 = 0
    for name in set2:
        ref_coor = ref_coors[name]
        dist2 += ((new_coor[0] - ref_coor[0]) ** 2 + (new_coor[1] - ref_coor[1]) ** 2) ** 0.5
    dist3 = 0
    for name in set3:
```

```
        ref_coor = ref_coors[name]
        dist3 += ((new_coor[0] - ref_coor[0]) ** 2 + (new_coor[1] - ref_coor[1]) ** 2) ** 0.5
    dist4 = 0
    for name in set4:
        ref_coor = ref_coors[name]
        dist4 += ((new_coor[0] - ref_coor[0]) ** 2 + (new_coor[1] - ref_coor[1]) ** 2) ** 0.5
    return [dist1, dist2, dist3, dist4]

[refs1, target_dist1] = get_dist(dot, data[sample]["sublima"])
[refs2, target_dist2] = get_dist(dot, data[sample]["ochracea"])
[refs3, target_dist3] = get_dist(dot, data[sample]["colorado"])
[refs4, target_dist4] = get_dist(dot, data[sample]["ssp"])
refs = [refs1, refs2, refs3, refs4]
dists = [target_dist1, target_dist2, target_dist3, target_dist4]
select_ind = dists.index(min(dists))

def get_deviation(coor):
    tgtdists = [target_dist1, target_dist2, target_dist3, target_dist4]
    newdists = get_residual(coor, refs1, refs2, refs3, refs4)
    ref_ind = tgtdists.index(min(tgtdists))
    Ntgtdists = []
    for dist in tgtdists:
        Ntgtdists.append(dist/tgtdists[ref_ind])
    Nnewdists = []
    for dist in newdists:
        Nnewdists.append(dist/newdists[ref_ind])
    deviation = 0
    for i in range(len(Ntgtdists)):
        if Nnewdists[i] > Ntgtdists[i]:
            deviation += Nnewdists[i] - Ntgtdists[i]
        else:
            deviation += Ntgtdists[i] - Nnewdists[i]
    return deviation

start_coor = [dot[1],dot[2]]
best_coor = minimize(get_deviation, start_coor, method='L-BFGS-B', tol=1e-10)
```

II.4. Analyzing the population structure using STRUCTURE (Pritchard, et al. 2000)

We first deduced the population structure for the 72 well-covered specimens (discussed above) using STRUCTURE. Then, we analyzed each of the 14 target specimens separately with a set of relatively pure specimens (4-6 specimens per subspecies, details in Table S4). Starting from the sequence alignments prepared for PCA, we removed positions that are close to each other in the genome. For each scaffold, the first position with SNPs was always taken, and the next one is only taken if it is at least 200 bp away from the previous position. This filtering further (in addition to the plink-based approach described above) removes positions that are tightly linked to each other. We selected the gap ratio (0.1, 0.15, 0.2 or 0.25)

that resulted in 50,000 - 100,000 positions, which is 0.15 for most cases. From the selected alignment, we generated 5 sets of input, and each set contains 50,000 randomly sampled positions.

We converted the SNPs into STRUCTURE input by encoding the two possible nucleotides (we only worked with biallelic loci) and missing data (or gap) as 0, 1, and -9, respectively. We represented the data for each specimen in two consecutive lines, and each line encodes one possible haplotype. We included 3 columns in each line before the SNP data, and these 3 columns encode LABEL, POPDATA and POPFLAG (these fields are explained in <https://www.ccg.unam.mx/~vinuesa/tlem09/docs/structure_doc.pdf>). The LABEL is the specimen ID. POPDATA indicates the subspecies each specimen belongs to based on other evidence (PCA, morphology and geography), and we used 1, 2, 3, 4, and 5 for specimens of *H. c. sublima*, *H. c. ochracea*, *H. c. idaho*, *H. c. colorado,* and those of hybrid origin, respectively. We do not want to use the POPDATA as priors for the analysis, and therefore we set POPFLAG to 0 for all the specimens.

In addition to the input SNP data, STRUCTURE needs two parameter files to explain the information in the input and control the STRUCTURE runs. The content of the mainparams file is as below.

```
#define MAXPOPS    4      // (int) number of populations assumed
#define BURNIN    20000   // (int) length of burnin period
#define NUMREPS   50000   // (int) number of MCMC reps after burnin
#define INFILE   infile   // (str) name of input data file
#define OUTFILE  outfile  //(str) name of output data file
#define NUMINDS    72 // (int) number of diploid individuals in data file
#define NUMLOCI     50000 // (int) number of loci in data file
#define PLOIDY    2 // (int) ploidy of data
#define MISSING    -9 // (int) value given to missing genotype data
#define ONEROWPERIND    0 // (B) store data for individuals in a single line
#define LABEL     1 // (B) Input file contains individual labels
#define POPDATA   1 // (B) Input file contains a population identifier
#define POPFLAG     1 // (B) Input file contains a flag which says whether to use popinfo when USEPOPINFO==1
#define LOCDATA    0 // (B) Input file contains a location identifier
#define PHENOTYPE 0    // (B) Input file contains phenotype information
#define EXTRACOLS 0    // (int) Number of additional columns before the genotype data start.
#define MARKERNAMES      0  // (B) data file contains row of marker names
#define RECESSIVEALLELES 0  // (B) data file contains dominant markers (eg AFLPs) and a row to indicate which alleles are recessive
#define MAPDISTANCES     0  // (B) data file contains row of map distances between loci
#define PHASED          0 // (B) Data are in correct phase (relevant for linkage model only)
#define PHASEINFO    0 // (B) the data for each individual contains a line indicating phase (linkage model)
#define MARKOVPHASE      0 // (B) the phase info follows a Markov model.
#define NOTAMBIGUOUS    -999 // (int) for use in some analyses of polyploid data
```

The content of the extraparams file is as below.

```
#define NOADMIX    0 // (B) Use no admixture model (0=admixture, 1=no-admix)
#define LINKAGE    0 // (B) Use the linkage model model
#define USEPOPINFO    0 // (B) Use prior population information to pre-assign individuals
#define LOCPRIOR    0 // (B) Use location information to improve weak data
```

#define FREQSCORR 1 // (B) Allele frequencies are correlated among pops
#define ONEFST 0 // (B) Assume same value of Fst for all subpopulations.
#define INFERALPHA 1 // (B) Infer ALPHA (the admixture parameter)
#define POPALPHAS 0 // (B) Individual alpha for each population
#define ALPHA 1.0 // (d) Dirichlet parameter for degree of admixture (the initial value if INFERALPHA==1).
#define INFERLAMBDA 0 // (B) Infer LAMBDA (the allele frequencies parameter)
#define POPSPECIFICLAMBDA 0 // (B) Infer a LAMBDA for each pop (if INFERLAMBDA=1).
#define LAMBDA 1.0 // (d) Dirichlet parameter for allele frequencies
#define FPRIORMEAN 0.01 // (d) Prior mean and SD of Fst for pops.
#define FPRIORSD 0.05 // (d) The prior is a Gamma distribution with these parameters
#define UNIFPRIORALPHA 1 // (B) Use a uniform prior for alpha; otherwise gamma prior
#define ALPHAMAX 10.0 // (d) Max value of alpha if uniform prior
#define ALPHAPRIORA 1.0 // Alpha has a gamma prior with mean A*B,
#define ALPHAPRIORB 2.0 // and variance A*B^2. (only if UNIFPRIORALPHA==0)
#define LOG10RMIN -4.0 // (d) Log10 of minimum allowed value of r under linkage model
#define LOG10RMAX 1.0 // (d) Log10 of maximum allowed value of r
#define LOG10RPROPSD 0.1 // (d) Standard deviation of log r in update
#define LOG10RSTART -2.0 // (d) Initial value of log10 r
#define GENSBACK 2 // (int) For use when inferring whether an individual is an immigrant, or has an immigrant ancestor in the past GENSBACK generations.
#define MIGRPRIOR 0.01 // (d) Prior prob that an individual is a migrant (used only when USEPOPINFO==1). This should be small, eg 0.01 or 0.1.
#define PFROMPOPFLAGONLY 0 // (B) Only use individuals with POPFLAG=1 to update P
#define LOCISPOP 1 // (B) Use POPDATA for location information
#define LOCPRIORINIT 1.0 //(d) initial value for r, the location prior
#define MAXLOCPRIOR 20.0 //(d) max allowed value for r
#define PRINTNET 1 // (B) Print the "net nucleotide distance" to screen during the run
#define PRINTLAMBDA 1 // (B) Print current value(s) of lambda to screen
#define PRINTQSUM 1 // (B) Print summary of current population membership to screen
#define SITEBYSITE 0 // (B) Whether or not to print site by site results (Linkage model only)
#define PRINTQHAT 0 // (B) Q-hat to a separate file. Turn this on before using STRAT.
#define UPDATEFREQ 100 // (int) Frequency of printing update on the screen.
#define PRINTLIKES 0 // (B) Print current likelihood to screen every rep
#define INTERMEDSAVE 0 // (int) Number of saves to file during run
#define ECHODATA 1 // (B) Print some of data file to screen
#define ANCESTDIST 0 // (B) Collect data about the distribution of Q for each individual
#define NUMBOXES 1000 // (int) Q stored as a histogram with this number of boxes.
#define ANCESTPINT 0.90 // (d) The size of the displayed probability interval on Q
#define COMPUTEPROB 1 // (B) Estimate the probability of the Data under the model
#define ADMBURNIN 500 // (int) Initial period of burnin with admixture model
#define ALPHAPROPSD 0.025 // (d) SD of proposal for updating alpha
#define STARTATPOPINFO 0 // Use given populations as the initial condition for population origins. (Need POPDATA==1).
#define RANDOMIZE 0 // (B) Use new random seed for each run
#define SEED 793870 // (int) Seed value for random number generator
#define METROFREQ 10 // (int) Frequency of using Metropolis step to update Q
#define REPORTHITRATE 0 // (B) report hit rate if using METROFREQ

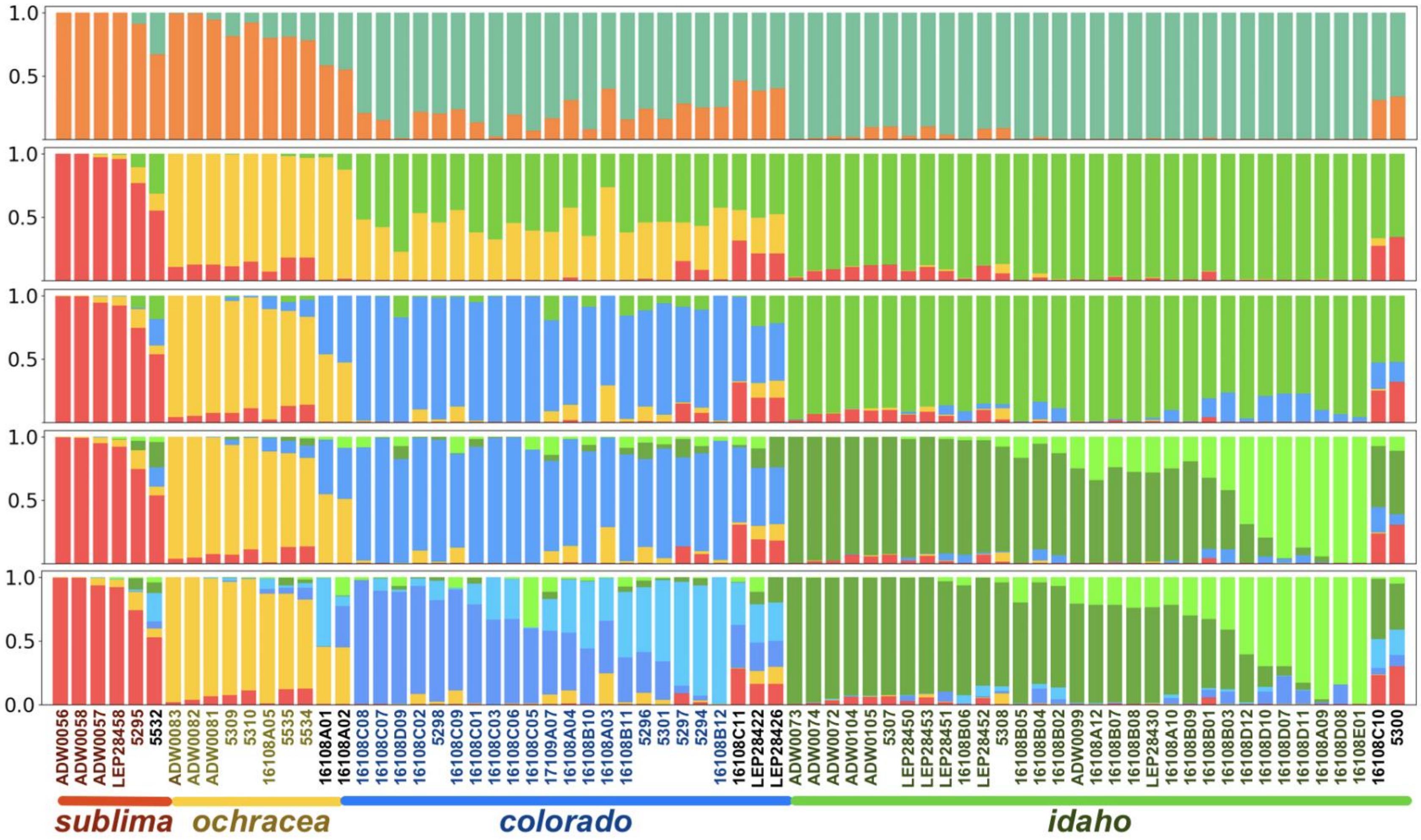

Fig. S12. STRUCTURE results for the 72 well-covered samples. Sample IDs and the subspecies they belong to are marked belong. The four panels from the top to the bottom represent results under different values of K (the number of populations): 2, 3, 4, 5, 6, respectively.

For each set of 50,000 positions (we have 5 sets in total), we ran STRUCTURE assuming 2, 3, 4, 5, 6 populations (K = 2, 3, 4, 5, 6) using the following command: structure -K [2, 3, 4, 5, or 6] -i [input] -o [output] >& log. For each set of positions, we used 10 replicates for STRUCTURE runs assuming 2, 3, 4, 5, and 6 populations, respectively. Each replicate was initiated with a different random seed. Therefore, for each value of K, we have 50 STRUCTURE runs: 5 datasets and 10 replicates for each dataset. A larger number of replicates is needed because the simulation may be trapped in some local minima, especially for larger K, and some replicates with probability much lower than the highest one need to be discarded. We inspected the STRUCTURE output with the highest probability ("Estimated Ln Prob" from the STRUCTURE output) for each K (Fig. S12). At K = 4, STRUCTURE produces results that show the highest probability (Fig. S13) and are consistent with the PCA and t-SNE results, suggesting four populations: *H. c. sublima*, *H. c. ochracea*, *H. c. colorado*, and *H. c. idaho*. When we assume a larger K, STRUCTURE will infer admixed ancestry for *H. c. colorado* and *H. c. idaho* specimens. When 3 populations are assumed, all *H. c. colorado* specimens are inferred as hybrids between *H. c. idaho* and *H. c. ochracea*. When K = 2, *H. c. sublima* is combined with *H. c. ochracea*, and *H. c. colorado* is merged with *H. c. idaho*.

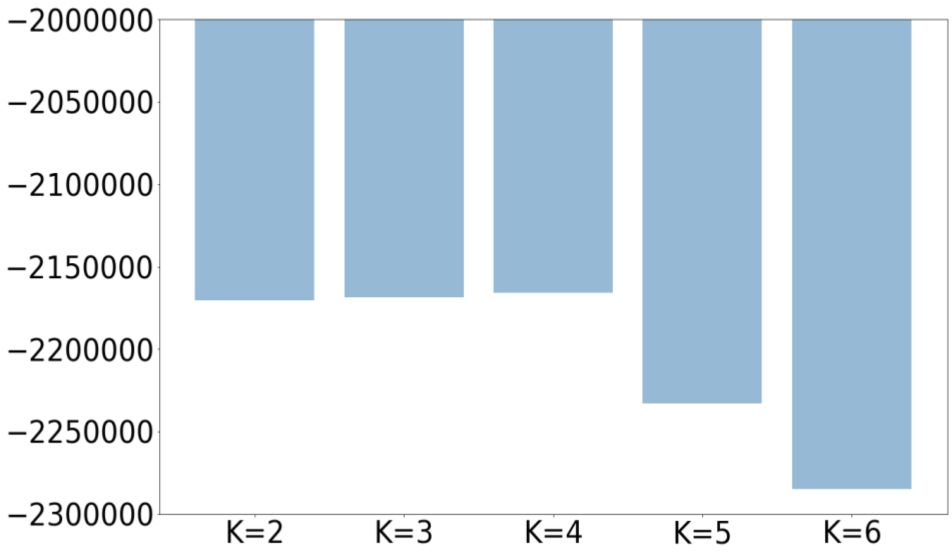

Fig. S13. The average estimated probability for the top 10% replicates with the highest probability for each assumed number of populations.

For each assumed number of populations (K), we took up to 10% of the STRUCTURE runs (5 out of 50) that generate the highest probability ("Estimated Ln Prob" from STRUCTURE output), and we excluded the replicates with probability lower than the highest one by more than 2%. The average probabilities of the selected replicates for different values of K are shown in Fig. S13. A population number of 4 receives the highest probability, and is thus the most probable inferred number of populations by STRUCTURE. In addition, we counted the number of hybrids (fraction of the dominant ancestry < 2/3) under each assumed number of populations (Fig. S14). When we assume 4 populations, there are the smallest number of hybrids. When we assume 2 populations, STRUCTURE combines *H. c. colorado* with *H. c. idaho*, and *H. c. sublima* with *H. c. ochracea*, and thus the number of inferred hybrids is comparable to that when we assume 4 populations. Therefore, the most likely number of populations inferred by STRUCTURE is four, and the ancestry for each specimen is consistent with the inference from morphology, geography, PCA, and t-SNE.

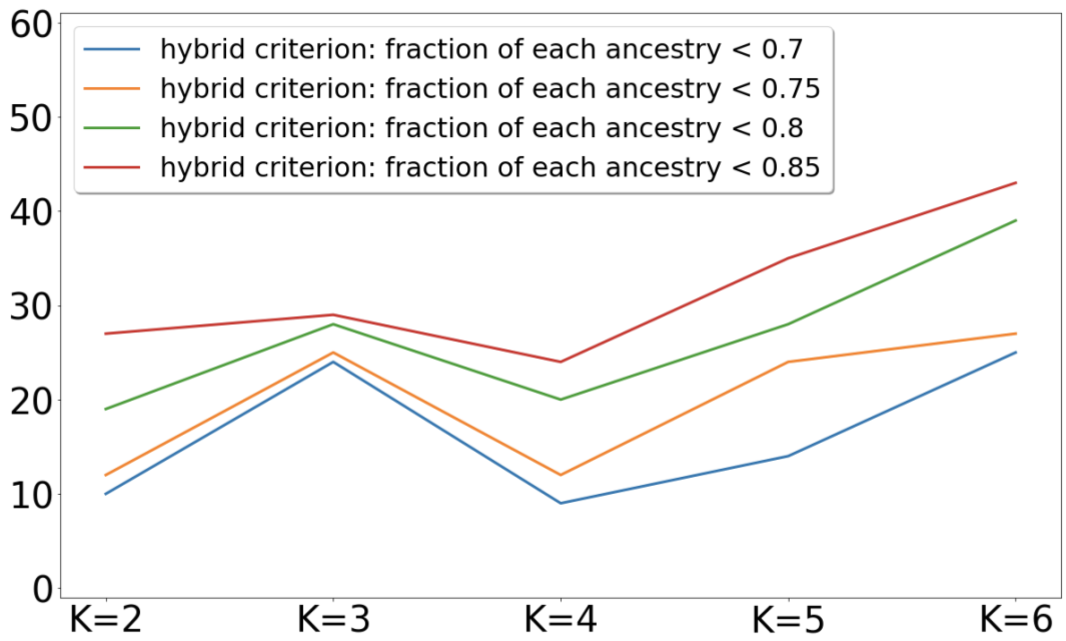

Fig. S14. Number of hybrid specimens under each assumed number of populations.

We assigned the ancestry of the target specimens using the relatively pure specimens (under K = 4) as reference. Specimens LEP-28458, ADW-0056, ADW-0057, and ADW-0058 were used as prototypes for *sublima*. Specimens NVG-5309, ADW-0081, ADW-0082, and ADW-0083 were used as prototypes for *ochracea.* Specimens NVG-5301, NVG-16108C02, NVG-16108B10, NVG-5298, and NVG-16108C07 were used as prototypes for *H. c. colorado.* Specimens ADW-0074, LEP-28430, NVG-16108D12, NVG-16108B07, ADW-0073, and ADW-0099 were used as prototypes for *idaho.* We added one target specimen at a time to the reference specimens, and processed the alignment as described above. One important difference was that for the reference specimens, we changed the POPFLAG from 0 to 1 to allow the program to use the pre-defined populations. For the target specimen that we wanted STRUCTURE to infer its ancestry, POPFLAG was still set to 0. In addition, we changed "#define NUMINDS 72" in the mainparams file to "#define NUMINDS 21" because there were 21 specimens (20 reference specimens and 1 target) being analyzed each time; we changed "#define USEPOPINFO 0" in the extraparams file to "#define USEPOPINFO 1" because we wanted to use the population information for the reference specimens.

The results of the STRUCTURE runs for individual target specimens are summarized in Fig. S15. Because the reference specimens were used as prototypes for the populations, their ancestry is 100% assigned to the population they belong to. NVG-5304 from Clear Creek County of Colorado state is inferred as a hybrid between *H. c. sublima* and *H. c. ochracea*, which is consistent with the result from PCA and the specimen locality at the boundary between the range of these two populations. NVG-6706 from Lake County of Colorado state is inferred as a hybrid between *H. c. colorado* and *H. c. sublima,* which is also consistent with its locality and the PCA result. All other specimens are assigned primarily to one population (subspecies). In particular, the lectotype (NVG-6313) and paralectotype (NVG-6708) of *H. c. colorado*, the Holotype (NVG-15111B01) and allotype (NVG-15111B02) of *H. c. ochracea* are all correctly assigned. In addition, STRUCTURE results were used to quantitatively define hybrids. We considered specimens with dominant ancestry consisting of less than 2/3 of their ancestry as hybrids. Therefore, the following

specimens were considered as hybrids: LEP-28422, LEP-28426, NVG-16108A01, NVG-16108A02, NVG-16108C10, NVG-16108C11, NVG-5300, NVG-5532, NVG-5304, and NVG-6706.

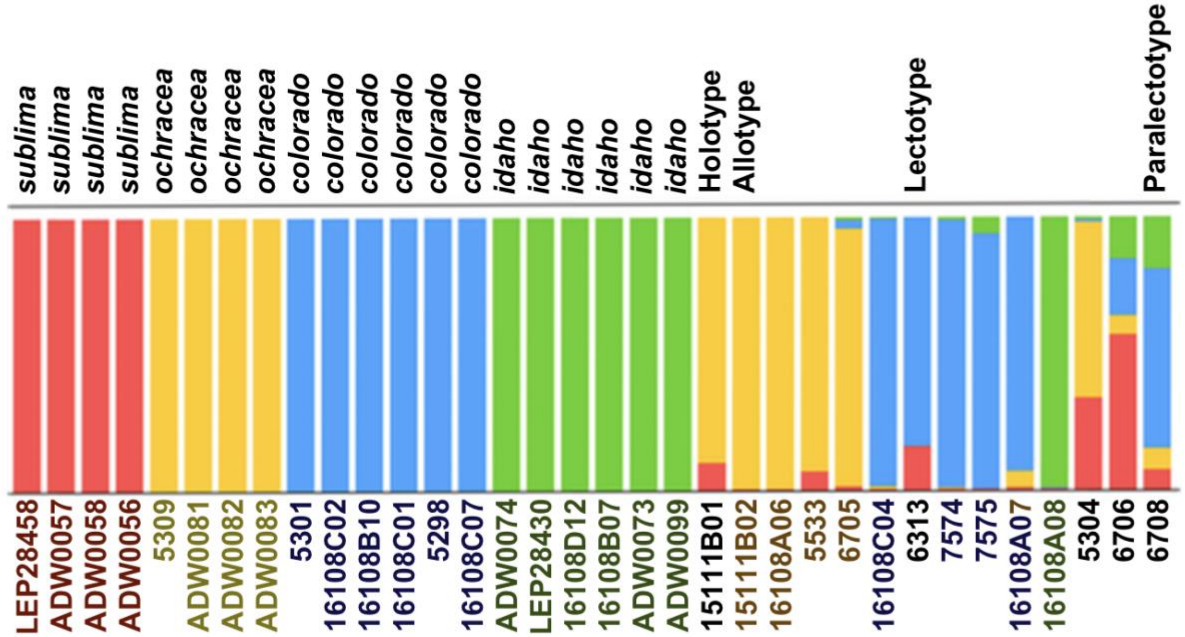

Fig. S15. Assigning the target specimens by STRUCTURE.

II.5. Analyzing the population structure using TREEMIX

According to our experience, Treemix is even less tolerant of gaps (missing data) in the input genotype data than PCA and STRUCTURE. We therefore tried to eliminate all the gaps in treemix input. To prepare the inputs of PCA and STRUCTURE, we used positions that are covered by at least two independent DNA fragments (not the forward and reverse read of the same fragment), and the rest were considered as gaps. However, to prepare the input for TreeMix, we relaxed this requirement to consider positions that were covered by just one DNA fragment. In addition, we used only the non-hybrid specimens with gap ratio less than 25% as backbone specimens to place the targets. A total of 51 specimens representing all four populations were selected (Table S4), and each of the remaining specimens was added to the backbone specimens to perform the analysis one by one. We processed the alignment to remove any positions with gaps and selected confident biallelic loci as described above. We obtained more than 200,000 positions in the alignment with the 51 backbone specimens. In order to obtain the support for each node, we selected 100 sets of 100,000 positions from each alignment for TreeMix analysis by random sampling (without return).

Since TreeMix works with the frequency of SNPs at each position, we used the number of reads supporting each of the two possible nucleotides (biallelic loci) in each specimen as input. For example, if a specimen is either A or T at a position, and A and T are supported by 4 and 6 reads, respectively, we encode the SNP frequencies at this position as 4,6 in the input for TreeMix. Below are the first 5 lines and first 10 columns of an input file for TreeMix. The first line marks which specimen each column corresponds to, and starting from the second line, each line consists of the SNP counts for populations at each selected position, where

different populations are separated by spaces, and the two alleles of each population are delimited by commas. Positions from the same scaffold are ordered by their location on the scaffold.

5307 5308 LEP28453 5294 5296 5297 5309 5310 5534 5535
3,0 1,0 3,0 5,2 3,3 5,0 1,0 3,0 6,0 8,0
5,0 2,1 4,0 5,2 3,3 3,2 2,0 1,1 6,0 8,0
0,1 0,5 0,3 0,3 0,4 0,4 0,1 0,4 0,5 0,4
1,0 0,5 0,3 0,3 0,4 0,4 0,1 0,3 0,4 0,4

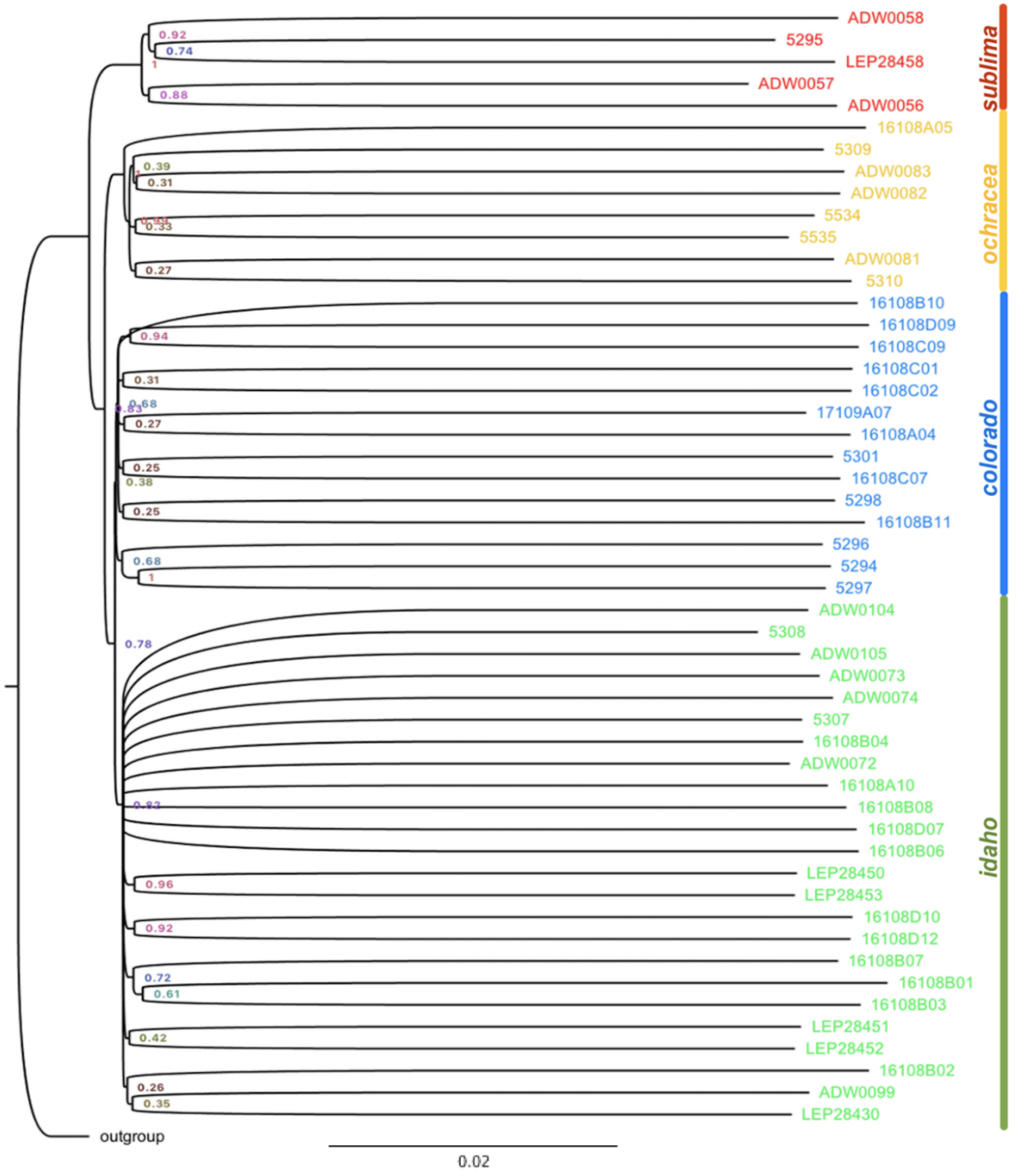

Fig. S16. Clades of subspecies and evolutionary history inferred by TreeMix. The support of each node is calculated as the number of random samples of the alignment that support this node. Nodes with support less than 0.25 are collapsed.

We used the following commands to run TreeMix on each of the 100 random samples of SNP data: gzip [input file]; treemix -i [input file].gz -o [output], and the 100 resulting trees were summarized by sumtrees.py <https://dendropy.org/programs/sumtrees.html> using the following command: sumtrees.py --suppress-annotations -f 0.25 [a file with all the 100 trees]. The resulting consensus tree was visualized in FigTree (Fig. S16). TreeMix again partitioned these specimens into four clades corresponding to the four subspecies. In addition, it suggested a possible evolutionary history of these subspecies: *sublima* branched out first, followed by *ochracea*, and finally *H. c. colorado* separated from *idaho.*

We next added the target specimens, one at a time, to the backbone specimens and repeated the above analysis. Including a target specimen to the alignment reduced the number of gap-free biallelic loci from over 200,000 to between 100,000 and 200,000 in most cases except NVG-6705 and NVG-6708. When there were more than 100,000 positions, we again obtained 100 random samples (without return) of 100,000 positions. When there were less than 100,000 positions, we performed 100 bootstrap sampling (with return) to get the same number of positions as that in the entire alignment. The analyses with one target specimen at a time reproduced the tree topology obtained from backbone specimens only, and they classified each target specimen into a subspecies by placing it within the clades corresponding to this subspecies. These assignments are again consistent with previous analyses, and the inferred subspecies assignment for each specimen by different methods are summarized in Table S5.

Section III: Phylogeny of *Hesperia colorado* and its sister species.

Hesperia colorado had been suggested to be a subspecies of *Hesperia comma*, and we therefore performed phylogenetic analysis using 45 representatives covering various subspecies of *Hesperia colorado* and *Hesperia comma* from a wide range (Fig. 2). Specimens from diverse localities and type specimens were preferred in our selection of representatives. One *Vernia verna* [formerly in *Pompeius*] specimen (NVG-18014H01) was added as the outgroup to root the tree.

Out of the selected specimens, NVG-6313 and NVG-6314 are the oldest and contain the largest amount of gaps. They are more likely to be affected by contamination and DNA damage with time, and thus we further cleaned up their sequences separately using the following protocol. We split the genomic regions that were not gap in the historical specimen into segments of no more than 100 bp. Within each 100 bp segment from the historical specimen, we count the number of positions where the nucleotide in this specimen is different from all other *Hesperia* specimens we have. If this number is larger than 1, we consider this fragment to be of poor quality and replace it with gaps. We then took positions that were not gap in either of these two historical specimens, and further removed positions that are gap in more than 20% of the selected specimens for phylogenetic analysis. A total of 1,911,543 positions from autosomes and 62,670 positions from the Z-chromosome passed these filters.

We obtained 100 random samples of 50,000 positions from the alignment of autosomal regions. For each sampled alignment, we performed phylogenetic analysis by IQ-TREE with the best substitution model

inferred by the program, TVM+F+R4 (Nguyen, et al. 2015). A consensus was derived from these trees using sumtrees.py <https://dendropy.org/programs/sumtrees.html>. Similarly, we did phylogenetic analysis on each of the 100 random samples of 50,000 positions from Z-chromosome with IQ-TREE (model: TVM+F+R3). Again, we used sumtree.py to get a consensus between the 100 trees on different samples. The mitochondrial genomes of these specimens contain very few gaps, with the highest gap fraction being 24%. We therefore did not filter the alignment (15,662 positions) and simply used bootstrap to generate 100 replicates and applied IQ-TREE on each replicate with the substitution model TIM2+F+R3.

Hesperia colorado and *Hesperia Colorado* form two monophyletic clades with a deep split between them, suggesting that they are different species. We tested this hypothesis using coalescent-based species delimitation method, Bayesian Phylogenetic & Phylogeography (BPP) (Flouri, et al. 2018). BPP employs Bayesian Markov chain Monte Carlo (MCMC) method to identify the parameters (including splitting of species) that maximize the likelihood of the observed sequences under multiple loci under the multispecies coalescent model. We selected one representative (the one with the least amount of gaps) for each of the *H. colorado* subspecies and each of the *H. comma* subspecies shown in Fig. 2. Thus, a total of 16 and 7 samples were selected for *H. colorado* and *H. comma*, respectively. In addition, *Hesperia nevada* and *Hesperia viridis* were added as the more distantly related species and the outgroup.

Although BPP allows gaps in the alignment, but inclusion of gaps appear to drastically decrease the speed of MCMC simulation and possibly affect the performance of the program because all the author-provided cases use gap-free alignments. We therefore used only gap-free positions and identified genomic segments (loci) consisting of 1000-2000 gap-free positions that are separated from each other by at least 10kb in the genome. We randomly selected 10 loci for each BPP simulation, and a total of 100 simulations were performed. The parameters in the "control" file used for BPP simulation are listed below. All simulations suggested a posterior probability of 100% for the hypothesis of *H. colorado* and *H. comma* being distinct species.

```
seed = -1
seqfile = Hesperia_bpp_colorado_comma.seq
Imapfile = Hesperia_bpp_colorado_comma.Imap
outfile = Hesperia_bpp_colorado_comma.out
mcmcfile = Hesperia_bpp_colorado_comma.mcmc
speciesdelimitation = 1 1 2 0.5
speciestree = 1
species&tree = 4 colorado comma nevada viridis
                 16 7 1 1
                 (((colorado,comma),nevada),viridis);
usedata = 1
nloci = 10
cleandata = 0
thetaprior = 3 0.003 e
tauprior = 3 0.01
finetune = 1: 0.01 0.0001 0.005 0.0005 0.2 0.01 0.01
print = 1 0 0 0
burnin = 100000
```

sampfreq = 10
nsample = 100000

In another project, we studied the imperial criteria for species delimitation using the pairs of butterfly populations or species across central TX suture zone, and we figured out that fixation indices and level of introgression (details described in https://www.biorxiv.org/content/10.1101/837666v1) computed on the sex chromosome can separate pairs of species from pairs of populations with a large gap in between (Cong, et al. 2019). We computed the same statistics for *H. comma* and *H. colorado*, and compared the values with those we obtained from pairs of conspecific populations or species we studied across central TX suture zone (Fig. S17). *H. comma* and *H. colorado* shows high fixation index and low level of introgression on Z-linked genes, similar to species pairs. Therefore, these imperial criteria also support the hypothesis of treating *H. colorado* and *H. comma* as different species.

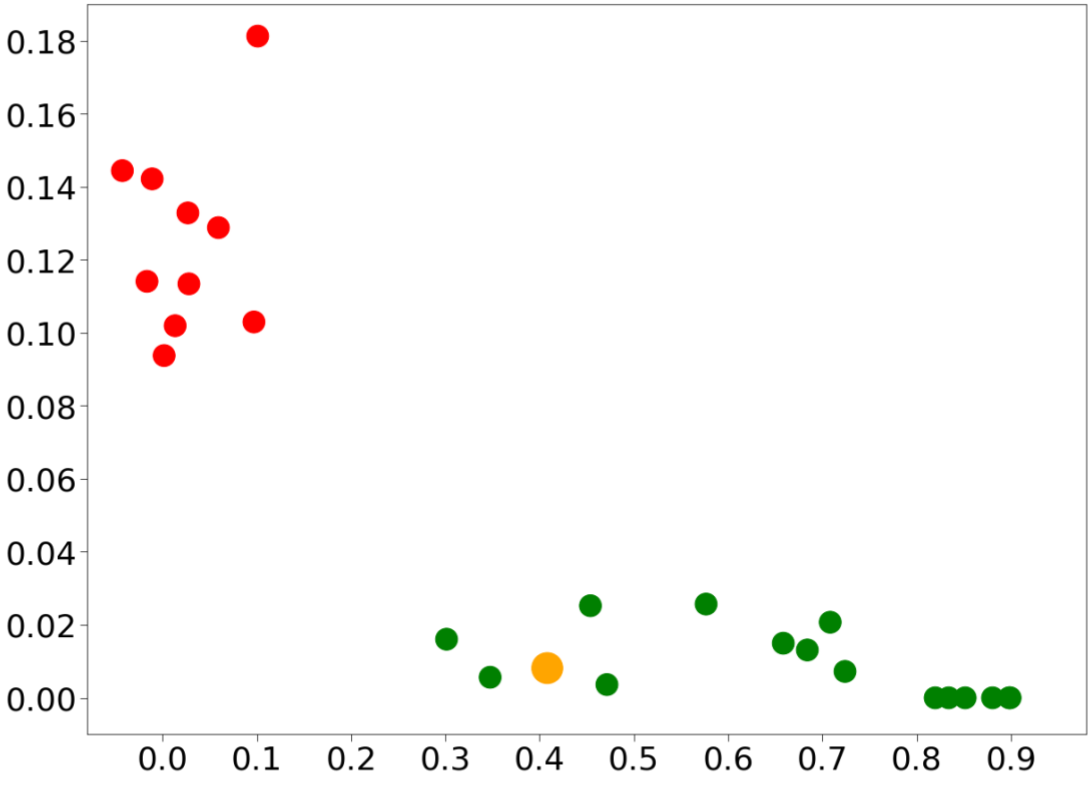

Fig. S17. Fixation indices (X-axis) and level of gene flow (Y-axis) computed on Z chromosomes. Red dots are pairs of conspecific populations and green dots are pairs of species across the central TX suture zone. Orange dot represents the pair of *H. colorado* and *H. comma*.

Section IV: Correlating the Genotype with Phenotype

IV.1. Investigating the high elevation adaptation in *H. c. sublima*

We selected five representative specimens for each subspecies: NVG-5295, ADW-0056, ADW-0057, ADW-0058, and LEP-28458 for *H. c. sublima*, NVG-5310, NVG-5534, NVG-5535, ADW-0081, and ADW-0082 for

H. c. ochracea, NVG-5294, NVG-5297, NVG-16108C02, NVG-16108C07, and NVG-17109A07 for *H. c. colorado*, NVG-5308, LEP-28450, LEP-28451, LEP-28452, and LEP-28453 for *H. c. idaho.* We identified the uniquely frequent SNPs for each subspecies represented by these specimens using the following criteria: (1) each subspecies has to contain 4 non-gap specimens at this position, (2) 75% of haplotypes from a subspecies contain a SNP that is absent in other *Hesperia colorado* subspecies (a) from the CO state (green bars) and (b) from worldwide (blue bars). Due to the limited sample size, some of these uniquely frequent SNPs are present in the ancestral population of *Heserpia colorado* at low frequency, and they became dominating as a result of genetic drift in a certain population. However, a significant fraction of these SNPs may be derived alleles originated as germ-line mutations, and their frequency was increased due to genetic drift or selective advantage. We further required the unique SNPs to be absent in *Hesperia comma*, and these SNPs are even more likely to be derived alleles resulted from mutation and genetic drift or selection.

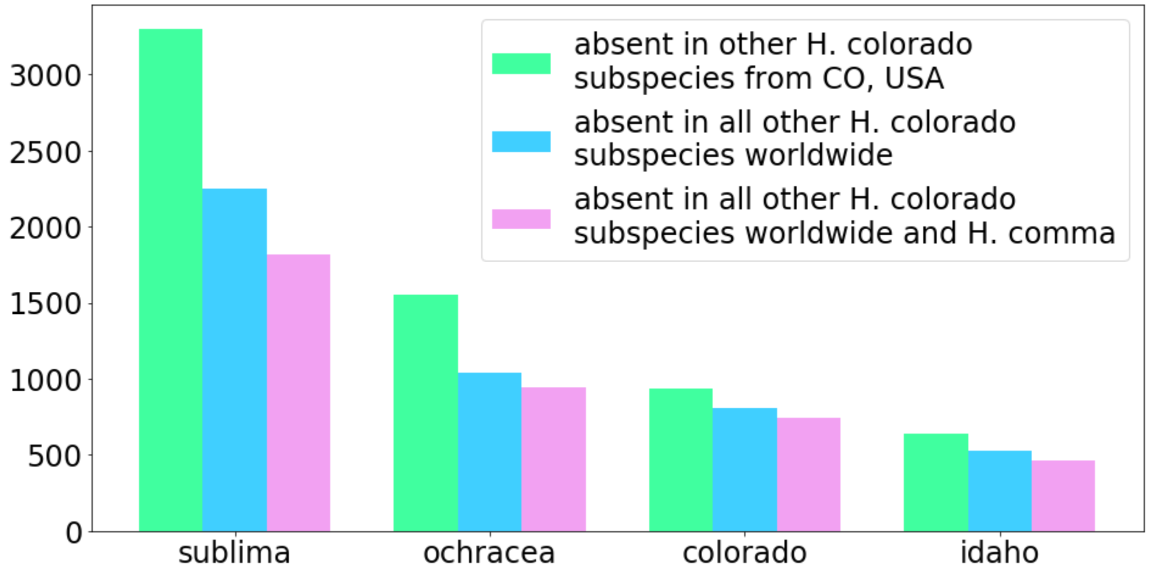

Fig. S18. Number of unique SNPs in each subspecies.

The number of uniquely frequent SNPs in each subspecies is shown in Fig. S18. The high elevation subspecies, *H. c. sublima* contains the highest number of unique positions, much higher than others. Two factors may contribute to the larger number of unique SNPs in *sublima*. First, *sublima* is relatively smaller population living on the mountain top, and therefore, genetic drift may be stronger in them. Indeed, *sublima* and specimens show the lowest level of population polymorphism (π) among the four Hesperia subspecies in the CO state (Fig. S19), indicating a smaller effective population size. The population polymorphism for *ochracea* is also much lower than the other two, which is again consistent with the relatively more restricted distribution of *ochracea* population and higher number of unique SNPs than *colorado* and *idaho*. In addition, *H. c. sublima* show the highest level of nonsynonymous mutation rate (number of nonsynonymous mutation/(number of synonymous and nonsynonymous mutations)) among the four subspecies, indicating stronger positive selection and possible positive selection in this mountain-top population. *H. c. ochracea* also exhibit much higher nonsynonymous mutation rate than the other two populations. *H. c. ochracea* live in different habitat (grass field) from other subspecies (stony mountain chains), and it is reasonable to expect some adaptation to the different environment.

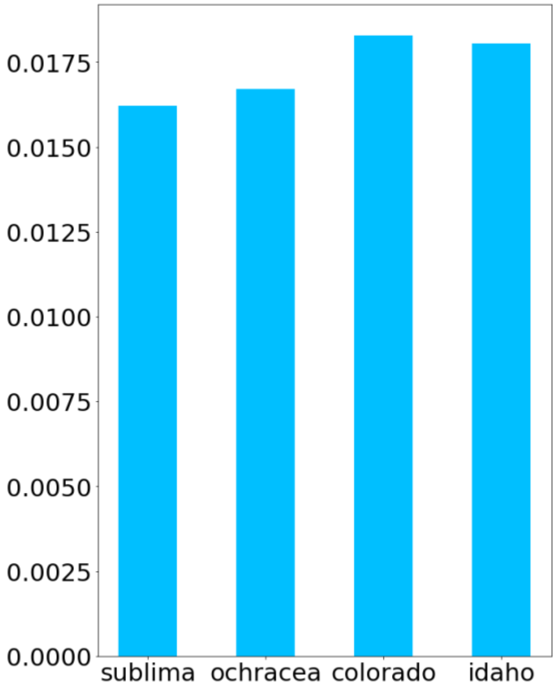

Fig. S19. Population polymorphism (π) for different *H. colorado* subspecies in the CO state.

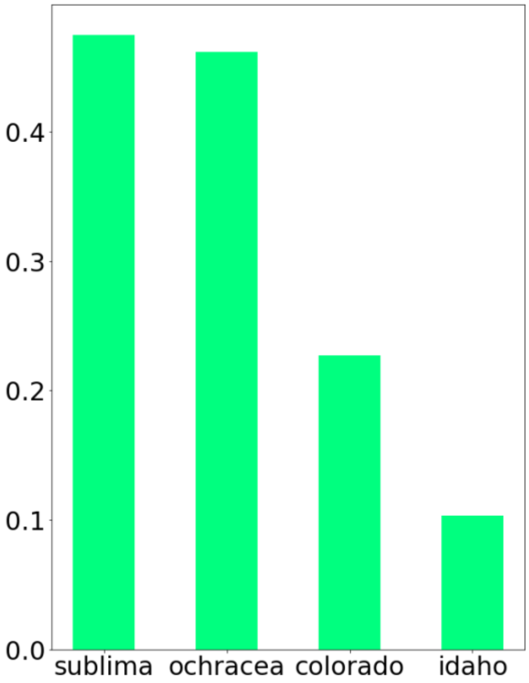

***Fig. S20.* Fraction of nonsynonymous ones among variations in the protein coding regions.**

We hypothesize that some of the *H. c. sublima*-specific SNPs may be related to its adaptation to living in the mountain top, where the weather is colder, windy and with a lower level of oxygen. We focused on the unique SNPs in the coding region, as it is hard to interpret the function of noncoding region and the impact of mutations happening in these regions. We identified 162 *sublima*-specific single amino acid variations by requiring their frequency in sublima to be at least 75% and frequency in low-elevation populations to be lower than 5%. We detected such SAVs in 80 proteins. These 80 proteins may be related to the high elevation adaptation of *sublima*. We analyzed their function using Gene Ontology (GO) (Gene Ontology Consortium 2008) terms. Enriched GO terms associated with these genes were detected using binomial tests (m = the number of proteins with high-elevation-specific SAVs that were associated with this GO term, N = number of proteins with high-elevation-specific SAVs, p = the probability for this GO term to be associated with any protein). GO terms with P-values lower than 0.01 were visualized using REVIGO (Supek, et al. 2011) and they are shown in Fig. S21.

In addition, we used BayeScan (Foll and Gaggiotti 2008) to detect candidate loci under positive selection based on frequencies of amino acids in the high-elevation subspecies and other low-elevation subspecies. BayeScan uses Markov chain Monte Carlo (MCMC) to test models with and without selection and estimates the posterior probability of observing the differences in allele frequencies between populations. It detects sites with positive or negative selection when the posterior probability with selection is statistically higher than the model without selection. BayeScan can correct the biases introduced by difference in the effective size and the immigration rate among populations, and it handles the uncertainty in allele frequencies caused by small samples sizes. BayeScan identified 118 sites from 72

proteins with significant sign of positive selection (Q-value < 0.1, which is means less than 10% of the predictions are expected to be false, this is a more stringent P-value that includes correction for multiple tests). Out of the 72 proteins with significant sign of positive selection, 48 overlap (P-value for significant overlap: 5e-324) are among the 80 proteins with *sublima*-specific high frequency SAVs, suggesting that these two methods can consistently identify candidates for positive selection. Similarly, we performed GO-term enrichment analysis on these 72 proteins with sign of positive selection, and the result is show in Fig. 3a. The most prominent GO-terms associated with proteins identified by both BayeScan and our criterion of *sublima*-specific SAVs are related to flight, muscle development, respiratory system development and sugar/lipid related metabolism.

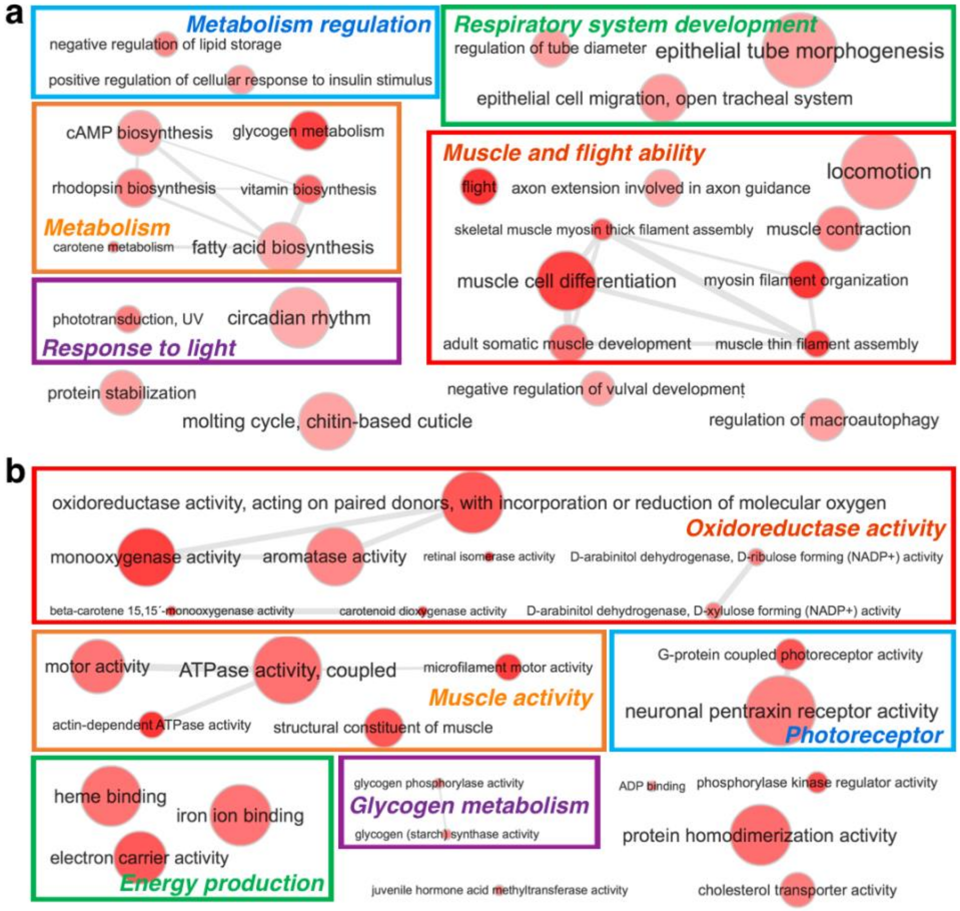

Fig. S21. Biological processes (a) and molecular functions (b) related to proteins with high-elevation-specific SAVs. The size of a circle correlates with the number of proteins in the genome associated with that GO term and its color indicates the significance (darker color corresponds to lower P-value) of a GO term enrichment among proteins with high-elevation-specific SAVs.

We also identified the positively selected genes in high-elevation subspecies (*H. c. sublima*) compared to the low-elevation subspecies (*H. c. colorado*, *H. c. ochracea*, and *H. c. idaho*) using McDonald-Kreitman (MK) tests (McDonald and Kreitman 1991). For high-elevation specimens, we estimated the number of nonsynonymous substitutions (P_N) and synonymous substitutions (P_S) needed to change from the codons of one specimen (A) to the codons of another specimen (B). If there were multiple substitution paths to change from the codon of A to the codon of B, the path with the smallest number of nonsynonymous substitutions (most parsimonious path) was taken. For each gene, P_N and P_S values for all pairs of high-elevation specimens were summed to get total P_N ($TP1_N$) and P_S ($TP1_S$). Similarly, we obtained the total

number of nonsynonymous substitutions ($TP2_N$) and synonymous substitutions ($TP2_S$) between all pairs of low-elevation specimens.

In addition, we obtained the dominant codon for high-elevation and low-elevation specimens, respectively. We used two different criteria to identify the dominant codons and performed the MK tests based on both of them, separately. In the first criterion, we considered codon in over 75% of specimens as the dominant one, and in the second criteria, we only considered changes that are completely fixed (present in all haplotypes). The original MK tests use changes that are completely fixed in each species to compute the interspecific nonsynonymous mutation rate (the second criterion). Here we also used a loosen criterion because we are studying subspecies that are not yet reproductively isolated. Divergence between subspecies may be on the way to become completely fixed and fixed changes may still exchange between subspecies as a result of occasional hybridization. Therefore, we used a loosen criterion to account for the changes that maybe selected but not yet fixed. We then counted the number of nonsynonymous substitutions (D_N) and synonymous substitutions (D_S) to change from the dominant codons of high-elevation specimens to the dominant codons of low-elevation specimens. To calculate the statistical significance for positive selection in each gene, we used binomial test ($p = (TP1_N + TP2_N) / (TP1_S + TP2_S + TP1_N + TP2_N)$, $N = D_S + D_N$, $m = D_N$) to evaluate whether D_N / D_S was significantly larger than $(TP1_N + TP2_N) / (TP1_S + TP2_S)$. A gene with P-value less than 0.05 was considered to be positively selected.

Interesting examples that are suggested to show significant sign of positive selection both by the allele frequency and the MK tests were studied manually. We searched for homologous 3D structures of these proteins in the Protein Data Bank (Burley, et al. 2017) using BLASTP (Altschul, et al. 1990), and the BLAST alignment was used to find the corresponding residue in the 3D structure for each residue in the query *Hesperia* protein.

```
Hesperia_colorado_sublima   DYILLRVENGIVVLEWNLGSGASRVSVDNIRVTDGYRHQVVVKLFYDNHVELEVDSEV
low_elevation               -----------------Q----------------------------------------
high_elevation              -----------------L----------------------------------------
Achalarus_lyciades          DFILLRVDRGIVVLEWNLGSGTASIALDSIVVTDGYRHQVIVKLYDDLHVELEVDRVT
Lerema_accius               DYILLLVENGVVVLEWNLGSGSSRVSVDNIRVTDGYHHMVVVKLFSDNHVELEVDREV
Papilio_glaucus             DYVLIRIENGVVVMEWDMGSGLNDIRIENVQVTDGERHEIIAKLMGDNNAYLSVDGVT
Papilio_machaon             DYVLIRIENGVVVMEWDMGSGLNEVRIENVHVTDGERHEIIAKLMTENQAYLSVDGTT
Phoebis_sennae              DFILIKIERGIVVMEWDLGSGLNSVAIDEVHVTDGENHNVIAKLFDDNHVELQVDNIS
Pieris_rapae                DYILIKVNRGLVEMEWDTGSGLNSIVIDGETVTDGESHNVIAKFYDVNRVELTVDGVS
Danaus_plexippus            DYVLLRVEGGAVVMEWDAGSGSNRIVIDDVLVTDGERHQIIVKFYEDRRVELNVDTVT
Libytheana_carinenta        DYIMIRVENGFVVMEWDLGSIKNTISIETVPVIDGDRHQIIAKLNADNRVELSVDSVS
Celastrina_neglecta         DFIMLRIEKGAVVMEWDVGSGPNSLSIPERRVTDGERHDVIAKLYGDMQVELTVDGIP
Eumaeus_atala               DYVLLRVDQGVVVLEWDTGSGQNQLIVEDITVTDGERHEVIFKLYEDSHVELIVDRVS
Calephelis_nemesis          DFIMLRVDQGRVLFEWNLGSGTNSVVCPEVPVNDGERHNVIVKLHEDGRVELWVDDYQ
```

Fig. S22. Alignment of a segment from motor neuron axon guidance factor *trol* with positions that differ between high-elevation and low-elevation subspecies.

IV.2. Genetic basis for the unique phenotype of *H. c. ochracea*

We analyzed the genomic distribution of the unique SNPs in each subspecies. While the unique SNPs for other subspecies are somewhat evenly distributed throughout the genome, 23% of the unique SNPs in *H.*

c. ochracea are concentrated in a 200 kbp region (0.03% of the entire genome) on the Z-chromosome out of the 609 Mbp genome. We constructed the phylogenetic tree for all the *Hesperia* samples we have using this 200 kb region. *H. c. ochracea* grouped with *H. c. assiniboia,* which distributes to the north of *H. c. ochracea*'s range. In addition, *H. c. ochracea* looks the most distinct from the other four *Hesperia colorado* subspecies we studied in the Colorado State, and instead it resembles the look of *H. c. assiniboia*. Therefore, we suspect that this 200 kbp region is introgressed from *H. c. assiniboia*, and we performed ABBA-BABA analysis to test our hypothesis.

ABBA-BABA test requires 4 taxa following a tree topology ((S1,S2),S3),O; where S1 and S2 are closely related, S3 is more distant and O is the outgroup. The test is used to identify introgression from a distant taxa S3 to either S1 or S2 based on excessive similarity between S3 and S1 or S2. In our case, O group includes all the *Hesperia comma* samples, S3 includes all the *H. c. assiniboia* samples*,* S1 is one of the four *Hesperia colorado* subspecies in CO state, and S2 includes the samples from the rest three *Hesperia colorado* subspecies in CO state. Therefore, we carried out ABBA-BABA tests in the following four setups: (1) ((*H. c. sublima*, *H. c. ochracea* + *H. c. colorado* + *H. c. idaho*), *H. c. assiniboia*), *H. comma*), (2) ((*H. c. ochracea*, *H. c. sublima* + *H. c. colorado* + *H. c. idaho*), *H. c. assiniboia*), *H. comma*), (3) ((*H. c. colorado*, *H. c. sublima* + *H. c. ochracea* + *H. c. idaho*), *H. c. assiniboia*), *H. comma*), (4) ((*H. c. idaho*, *H. c. sublima* + *H. c. ochracea* + *H. c. colorado*), *H. c. assiniboia*), *H. comma*).

We divided the genome into 200 kb windows. For each 200 kb window in each setup, we counted the number of positions following the pattern of ABBA or BABA in taxa S1, S2, S3, and O. A pattern of ABBA means that taxa S1 and O share the same nucleotide, and taxa S2 and S3 share the same nucleotide that is different from S1. A pattern of BABA means that taxa S2 and O share the same nucleotide, and taxa S1 and S3 share the same nucleotide that is different from S2. We used multiple specimens for each taxon, and different specimens may not support the same pattern. Therefore, we enumerated all possible specimens from the four taxa, and counted the fraction of enumerations that support a certain pattern at each position. We summed up the numbers we got for each position in the 200 kbp window to obtain the total number of ABBA positions and BABA positions, respectively. The difference between the number of BABA positions and the number of ABBA positions is expected to be 0 if there is no introgression. A value significantly larger than 0 suggests introgression from *H. c. assiniboia.* We plotted the difference between BABA and ABBA positions for each setup in genomic windows throughout the genome. The same 200 kb window discussed above shows the highest BABA - ABBA value in the test where *H. c. ochracea* is used as S1, much higher than any other genomic windows or when any other subspecies was tested.

References

Altschul SF, Gish W, Miller W, Myers EW, Lipman DJ. 1990. Basic local alignment search tool. J Mol Biol 215:403-410.
Bao W, Kojima KK, Kohany O. 2015. Repbase Update, a database of repetitive elements in eukaryotic genomes. Mob DNA 6:11.
Bolger AM, Lohse M, Usadel B. 2014. Trimmomatic: a flexible trimmer for Illumina sequence data. Bioinformatics 30:2114-2120.

Burley SK, Berman HM, Kleywegt GJ, Markley JL, Nakamura H, Velankar S. 2017. Protein Data Bank (PDB): The Single Global Macromolecular Structure Archive. Methods Mol Biol 1607:627-641.
Cong Q, Borek D, Otwinowski Z, Grishin NV. 2015a. Skipper genome sheds light on unique phenotypic traits and phylogeny. BMC Genomics 16:639.
Cong Q, Borek D, Otwinowski Z, Grishin NV. 2015b. Tiger swallowtail genome reveals mechanisms for speciation and caterpillar chemical defense. Cell Reports 10:910–919.
Cong Q, Grishin NV. 2016. The complete mitochondrial genome of Lerema accius and its phylogenetic implications. PeerJ 4:e1546.
Cong Q, Shen J, Borek D, Robbins RK, Otwinowski Z, Grishin NV. 2016. Complete genomes of hairstreak butterflies, their speciation, and nucleo-mitochondrial incongruence. Scientific Reports 6:24863.
Cong Q, Shen J, Li W, Borek D, Otwinowski Z, Grishin NV. 2017. The first complete genomes of metalmarks and the classification of butterfly families. Genomics 109:485–493.
Cong Q, Zhang J, Grishin NV. 2019. Genomic determinants of speciation. bioRxiv:837666.
dos Santos G, Schroeder AJ, Goodman JL, Strelets VB, Crosby MA, Thurmond J, Emmert DB, Gelbart WM, FlyBase C. 2015. FlyBase: introduction of the Drosophila melanogaster Release 6 reference genome assembly and large-scale migration of genome annotations. Nucleic Acids Res 43:D690-697.
Flouri T, Jiao X, Rannala B, Yang Z. 2018. Species Tree Inference with BPP Using Genomic Sequences and the Multispecies Coalescent. Mol Biol Evol 35:2585-2593.
Foll M, Gaggiotti O. 2008. A genome-scan method to identify selected loci appropriate for both dominant and codominant markers: a Bayesian perspective. Genetics 180:977-993.
Fraisse C, Picard MAL, Vicoso B. 2017. The deep conservation of the Lepidoptera Z chromosome suggests a non-canonical origin of the W. Nat Commun 8:1486.
Gene Ontology Consortium. 2015. Gene Ontology Consortium: going forward. Nucleic Acids Res 43:D1049-1056.
Gene Ontology Consortium. 2008. The Gene Ontology project in 2008. Nucleic Acids Res 36:D440-444.
Haas BJ, Salzberg SL, Zhu W, Pertea M, Allen JE, Orvis J, White O, Buell CR, Wortman JR. 2008. Automated eukaryotic gene structure annotation using EVidenceModeler and the Program to Assemble Spliced Alignments. Genome Biol 9:R7.
Heliconius Genome Consortium. 2012. Butterfly genome reveals promiscuous exchange of mimicry adaptations among species. Nature 487:94–98.
Kajitani R, Toshimoto K, Noguchi H, Toyoda A, Ogura Y, Okuno M, Yabana M, Harada M, Nagayasu E, Maruyama H, et al. 2014. Efficient de novo assembly of highly heterozygous genomes from whole-genome shotgun short reads. Genome Res 24:1384-1395.
Kawamoto M, Jouraku A, Toyoda A, Yokoi K, Minakuchi Y, Katsuma S, Fujiyama A, Kiuchi T, Yamamoto K, Shimada T. 2019. High-quality genome assembly of the silkworm, Bombyx mori. Insect Biochem Mol Biol 107:53-62.
Kelley DR, Schatz MC, Salzberg SL. 2010. Quake: quality-aware detection and correction of sequencing errors. Genome Biol 11:R116.
Korf I. 2004. Gene finding in novel genomes. BMC Bioinformatics 5:59.
Li H, Durbin R. 2009. Fast and accurate short read alignment with Burrows-Wheeler transform. Bioinformatics 25:1754-1760.
Li H, Handsaker B, Wysoker A, Fennell T, Ruan J, Homer N, Marth G, Abecasis G, Durbin R, Genome Project Data Processing S. 2009. The Sequence Alignment/Map format and SAMtools. Bioinformatics 25:2078-2079.
Li W, Cong Q, Shen J, Zhang J, Hallwachs W, Janzen DH, Grishin NV. 2019. Genomes of skipper butterflies reveal extensive convergence of wing patterns. Proceedings of the National Academy of Sciences of the United States of America 116:6232–6237.

Li X, Fan D, Zhang W, Liu G, Zhang L, Zhao L, Fang X, Chen L, Dong Y, Chen Y, et al. 2015. Outbred genome sequencing and CRISPR/Cas9 gene editing in butterflies. Nat Commun 6:8212.
Lomsadze A, Ter-Hovhannisyan V, Chernoff YO, Borodovsky M. 2005. Gene identification in novel eukaryotic genomes by self-training algorithm. Nucleic Acids Res 33:6494-6506.
McDonald JH, Kreitman M. 1991. Adaptive protein evolution at the Adh locus in Drosophila. Nature 351:652-654.
Nguyen LT, Schmidt HA, von Haeseler A, Minh BQ. 2015. IQ-TREE: a fast and effective stochastic algorithm for estimating maximum-likelihood phylogenies. Mol Biol Evol 32:268-274.
Pickrell JK, Pritchard JK. 2012. Inference of population splits and mixtures from genome-wide allele frequency data. PLoS Genet 8:e1002967.
Price AL, Patterson NJ, Plenge RM, Weinblatt ME, Shadick NA, Reich D. 2006. Principal components analysis corrects for stratification in genome-wide association studies. Nat Genet 38:904-909.
Pritchard JK, Stephens M, Donnelly P. 2000. Inference of population structure using multilocus genotype data. Genetics 155:945-959.
Purcell S, Neale B, Todd-Brown K, Thomas L, Ferreira MA, Bender D, Maller J, Sklar P, de Bakker PI, Daly MJ, et al. 2007. PLINK: a tool set for whole-genome association and population-based linkage analyses. Am J Hum Genet 81:559-575.
Shen J, Cong Q, Borek D, Otwinowski Z, Grishin NV. 2017. Complete genome of *Achalarus lyciades*, the first representative of the Eudaminae subfamily of skippers. Current Genomics 18:366–374.
Shen J, Cong Q, Kinch LN, Borek D, Otwinowski Z, Grishin NV. 2016. Complete genome of *Pieris rapae*, a resilient alien, a cabbage pest, and a source of anti-cancer proteins. F1000Research 5:2631.
Slater GS, Birney E. 2005. Automated generation of heuristics for biological sequence comparison. BMC Bioinformatics 6:31.
Stanke M, Steinkamp R, Waack S, Morgenstern B. 2004. AUGUSTUS: a web server for gene finding in eukaryotes. Nucleic Acids Res 32:W309-312.
Supek F, Bosnjak M, Skunca N, Smuc T. 2011. REVIGO summarizes and visualizes long lists of gene ontology terms. PLoS One 6:e21800.
Thurmond J, Goodman JL, Strelets VB, Attrill H, Gramates LS, Marygold SJ, Matthews BB, Millburn G, Antonazzo G, Trovisco V, et al. 2019. FlyBase 2.0: the next generation. Nucleic Acids Res 47:D759-D765.
Trapnell C, Pachter L, Salzberg SL. 2009. TopHat: discovering splice junctions with RNA-Seq. Bioinformatics 25:1105-1111.
Trapnell C, Williams BA, Pertea G, Mortazavi A, Kwan G, van Baren MJ, Salzberg SL, Wold BJ, Pachter L. 2010. Transcript assembly and quantification by RNA-Seq reveals unannotated transcripts and isoform switching during cell differentiation. Nat Biotechnol 28:511-515.
UniProt Consortium. 2019. UniProt: a worldwide hub of protein knowledge. Nucleic Acids Res 47:D506-D515.
van der Maaten L, Hinton G. 2008. Visualizing Data using t-SNE. Journal of Machine Learning Research 9:2579-2605.
Zhan S, Merlin C, Boore JL, Reppert SM. 2011. The monarch butterfly genome yields insights into long-distance migration. Cell 147:1171-1185.
Zhang J, Cong Q, Shen J, Brockmann E, Grishin NV. 2019. Genomes reveal drastic and recurrent phenotypic divergence in firetip skipper butterflies (Hesperiidae: Pyrrhopyginae). Proceedings of the Royal Society B: Biological Sciences 286:20190609.
Zhang J, Kobert K, Flouri T, Stamatakis A. 2014. PEAR: a fast and accurate Illumina Paired-End reAd mergeR. Bioinformatics 30:614-620.
